# Development of Noval, Recyclable, and Tunable Aryl Imidazolium Salts with a Dual Brønsted–Lewis Acid Character as Catalysts for the Copper(I)-Catalyzed Azide–Alkyne Cycloaddition Reaction (CuAAC)

**DOI:** 10.1021/acsorginorgau.6c00066

**Published:** 2026-08-13

**Authors:** Wen-Yueh Ho, Yu-Hsuan Yang, Jyun-Siao Chen, Ratnnadeep C. Sawant, Tzu-Hsin Shu, Pei-Yu Chen, Chia-Yun Chang, Shun-Yuan Luo

**Affiliations:** † Department of Food & Drug Industry and Inspective Technology, 71664 Chia Nan University of Pharmacy and Science, Tainan 717, Taiwan; ‡ Department of Chemistry, 34916 National Chung Hsing University, Taichung 402, Taiwan; § Department of Chemistry, 565285 Dr. Ambedkar College, Deekshabhoomi, Nagpur 440010, India

**Keywords:** CuAAC (copper(I)-catalyzed azide−alkyne cycloaddition), aryl imidazolium salts, dual Brønsted–Lewis acid, recyclable catalysts, click chemistry

## Abstract

The development of novel aryl imidazolium salts (ISs) with dual Brønsted–Lewis acid character represents a significant advancement in the field of green chemistry and catalysis. These imidazolium salts serve as efficient catalysts for the copper­(I)-catalyzed azide–alkyne cycloaddition (CuAAC) reaction, a cornerstone of click chemistry. The unique design of the aryl imidazolium framework, incorporating both Brønsted and Lewis acid sites, enhances the catalytic efficiency and selectivity of the CuAAC reaction. Furthermore, the use of these imidazolium salts as catalysts eliminates the need for additional phase-transfer catalysts or cosolvents, simplifying the reaction setup and reducing environmental impact. This study demonstrates the synthesis, characterization, and application of these novel imidazolium salts, highlighting their potential as sustainable and efficient media for CuAAC reactions. The developed catalyst is stable in water and air, and the highest catalytic activity could still be maintained after 4 cycles. The results indicate that the novel, recyclable, and tunable aryl imidazolium salts with dual Brønsted–Lewis acid properties offer a promising platform for the development of greener and more efficient catalytic processes in organic synthesis.

## Graphical abstract

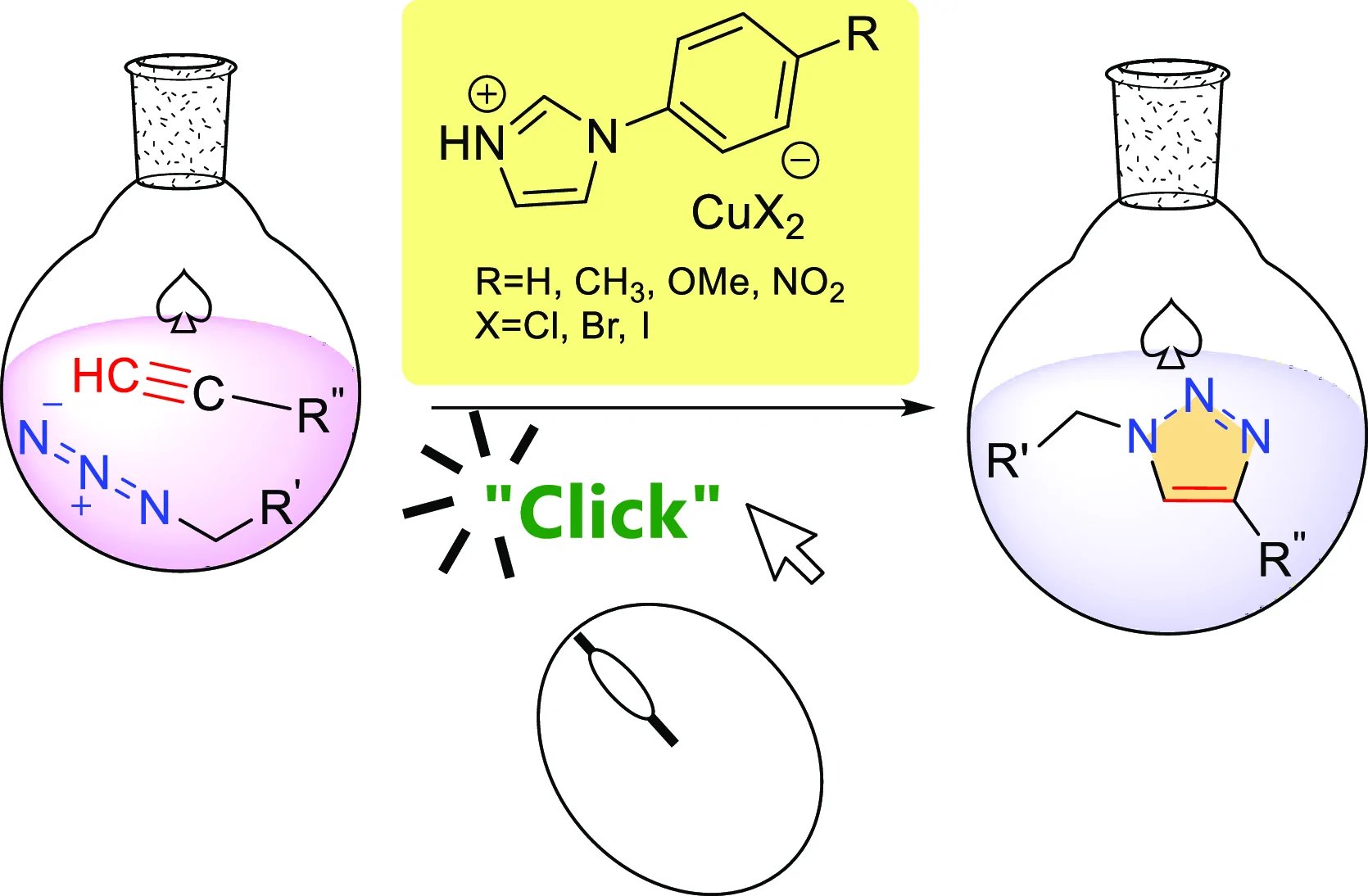

## Introduction

In an uncatalyzed reaction, the azide–alkyne Huisgen cycloaddition of organic azides and alkynes is a slow, high-temperature, and nonselective reaction, which produces an equimolar mixture of 1,4- and 1,5-disubstituted triazoles (Scheme 1 A).^1–3^ However, since the copper­(I)-catalyzed azide–alkyne cycloaddition (CuAAC) discovery in 2002 by the groups of Sharpless, Forkin, and Meldal independently, which are highly efficient, straightforward, and mild procedures for the regioselective synthesis of 1,4-disubstituted triazoles (Scheme 1B).^4^ This technique has received wide applications in such diverse fields as bioconjugation, polymer ligation, organic and medicinal syntheses, and surface science.^5–16^

**Scheme 1:**
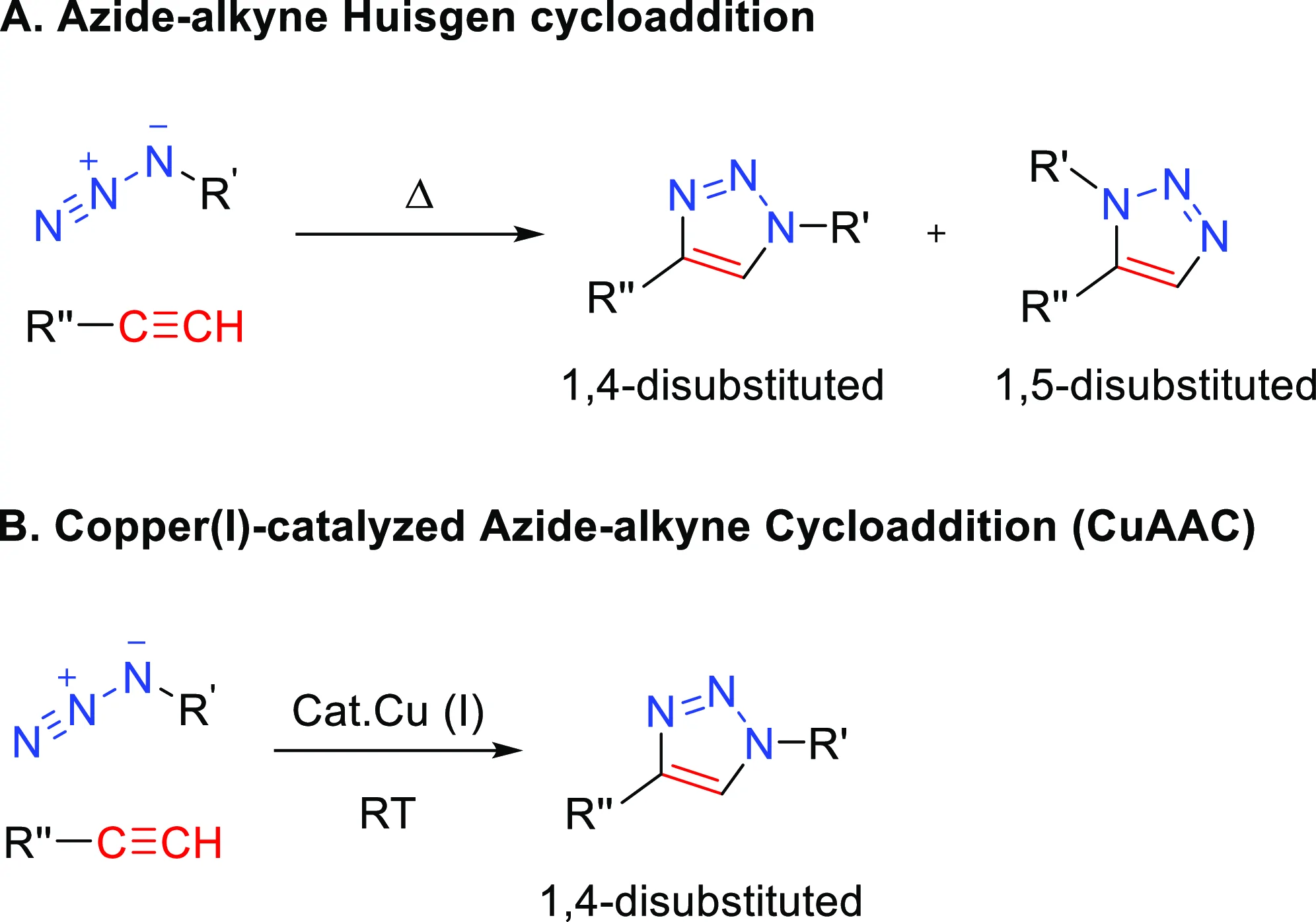
Synthesis of 1,2,3-Triazoles via Huisgen Cycloaddition and CuAAC

Over the past few decades, a great variety of copper precursors have been used in the CuAAC reaction.^17–36^ Recently, transition-metal-based molten salts have caught the attention of chemists.^37–39^ In this work, because the organic substrates and the synthesized aryl imidazolium salt catalyst are insoluble or only partially soluble in water, the system operates as a heterogeneous “on-water” catalytic system. In comparison with other reported catalysts, this heterogeneous mixture avoids the use of organic cosolvents and offers notable advantages in ease of preparation and direct recyclability via centrifugation, allowing the reaction to proceed smoothly at the solid–liquid–liquid interface under mild conditions.

While current research on imidazole-based molten salts predominantly focuses on platforms bearing sp^3^-hybridized alkyl substituents, introducing aryl substituents with distinct electron-withdrawing or electron-donating groups onto the imidazole core can significantly modulate the physicochemical features of the resulting ionic matrix via mesomeric effects. Considering the importance of these attractive properties and in continuation of our experience in molten salt catalysis.^40–44^ Herein, we report the application of Cu­(I) in molten salts as a reusable catalyst system. This study investigated catalyzed-CuAAC reactions of different substituents in aryl and distinct anions as a ligand with copper­(I) in molten salts. In comparison with other reported catalysts, this catalyst offers notable advantages in ease of preparation and recyclability. Additionally, it enables the reaction to proceed smoothly under mild conditions—namely, at room temperature and ambient pressure—with water serving as an environmentally friendly solvent.^45–52^ Besides, we also explored the structure–activity relationship based on molten salts’ physical and chemical properties.

## Discussion

According to the previously developed iron metal-based aryl imidazolium salts used in Friedel–Crafts reactions and thioesterification.^41,53^ This study that was an iron anion of molten salt was changed into a copper-based anion and was used as a novel catalyst in the click reaction. The synthesis of Cu-based imidazolium salts is summarized in Scheme 2. First, structure **A** was dissolved in ethanol and neutralized with hydrohalic acid in an ice bath to produce Brønsted acidic imidazolium salt **B**. In the last step, the halide ions in **B** were coordinated with copper­(I) halide in ethanol to obtain **IS-Cl1** to **IS-I4** in good yields. The structural integrity and purity of the synthesized aryl imidazolium salts were confirmed by elemental analysis and high-resolution mass spectrometry (HRMS). The elemental analysis (EA) results for C, H, and N were in good agreement with the calculated values for the proposed molecular formulas, supporting the high purity of the isolated products. In addition, HRMS analysis showed the expected molecular ion peaks corresponding to the imidazolium cations, further confirming the successful synthesis of the target compounds. Detailed analytical data for each imidazolium salts are provided in the Experiments Section and Supporting Information (Tables S1 and S2). This synthetic route demonstrates an atom economy of close to 100%. Since substituents on the aromatic rings of the imidazolium salts are not restricted to van der Waals interactions but also enable π–π interactions. These effects are important for the stabilization of catalytically active metals.^54^

**Scheme 2:**
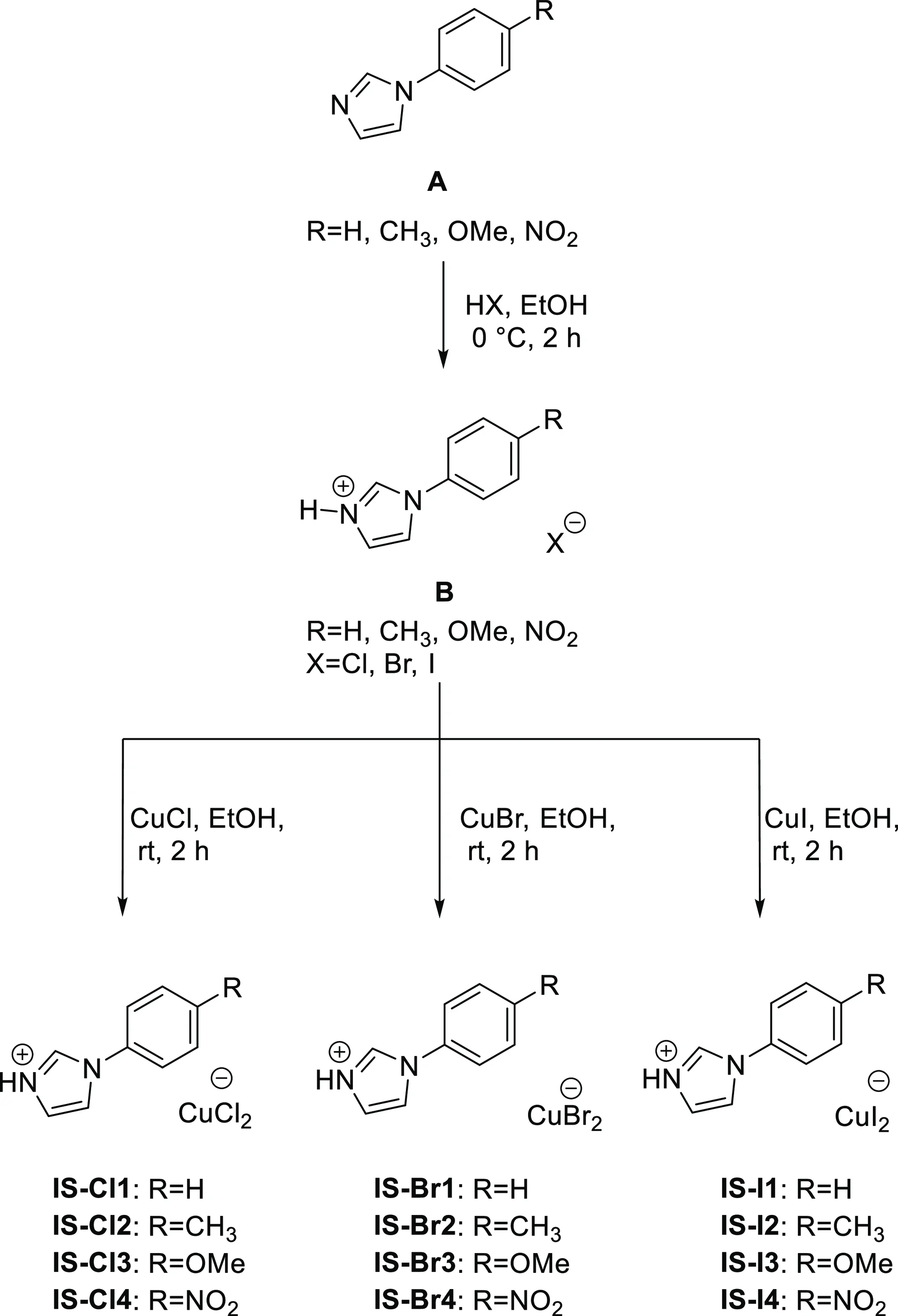
Synthesis of Dual Acidic Tunable Aryl Imidazolium Salts (DATAISs: IS-Cl1 to IS-I4)

To study the correlation between the acidity of individual imidazolium salts, pyridine is used for confirmation of the Lewis and Brønsted acidities through monitoring the bands in the range of 1400–1700 cm^–1^ arising from its vibration modes.^55^ As the DATAISs have Lewis acidity as a content [CuX_2_]^−^ complex anion, whose acidity could be affected by a ligand of anion. Hence, the Lewis acidity of the DATAISs could be measured with FTIR. Figure 1 shows the FTIR spectra of the DATAISs with a pyridine base on the same aryl imidazolium cation. Since the previous study expressed that pyridine, added to the [BMIM]­Cl/FeCl_3_ imidazolium salt results in the band, would upfield from 1437 cm^–1^.^56^ Based on the shift of this band, Lewis acidity anions ([CuX_2_]^−^) increase in the order [CuCl_2_]^−^ > [CuBr_2_]^−^ > [CuI_2_]^−^. Besides, the presence of the band near 1540 cm^–1^ is an indication of the pyridinium-ion formation. The Brønsted acid for the DATAISs in the N atom and C2-imidazolium in the aryl imidazolium moiety has proton bonding, causing the effect. Moreover, the Brønsted acidity effect’s strength depends on the substituent groups on the aromatic group. According to the IR spectrum of **IS-I1** to **IS-I4**, it was found that Brønsted acidity increases in the order **IS-I3** (R = OCH_3_, 1522 cm^–1^) > **IS-I2** (R = CH_3_, 1519 cm^–1^) > **IS-I1** (R = H, 1515 cm^–1^) > **IS-I4** (R = NO_2_, 1512 cm^–1^).

**Figure 1:**
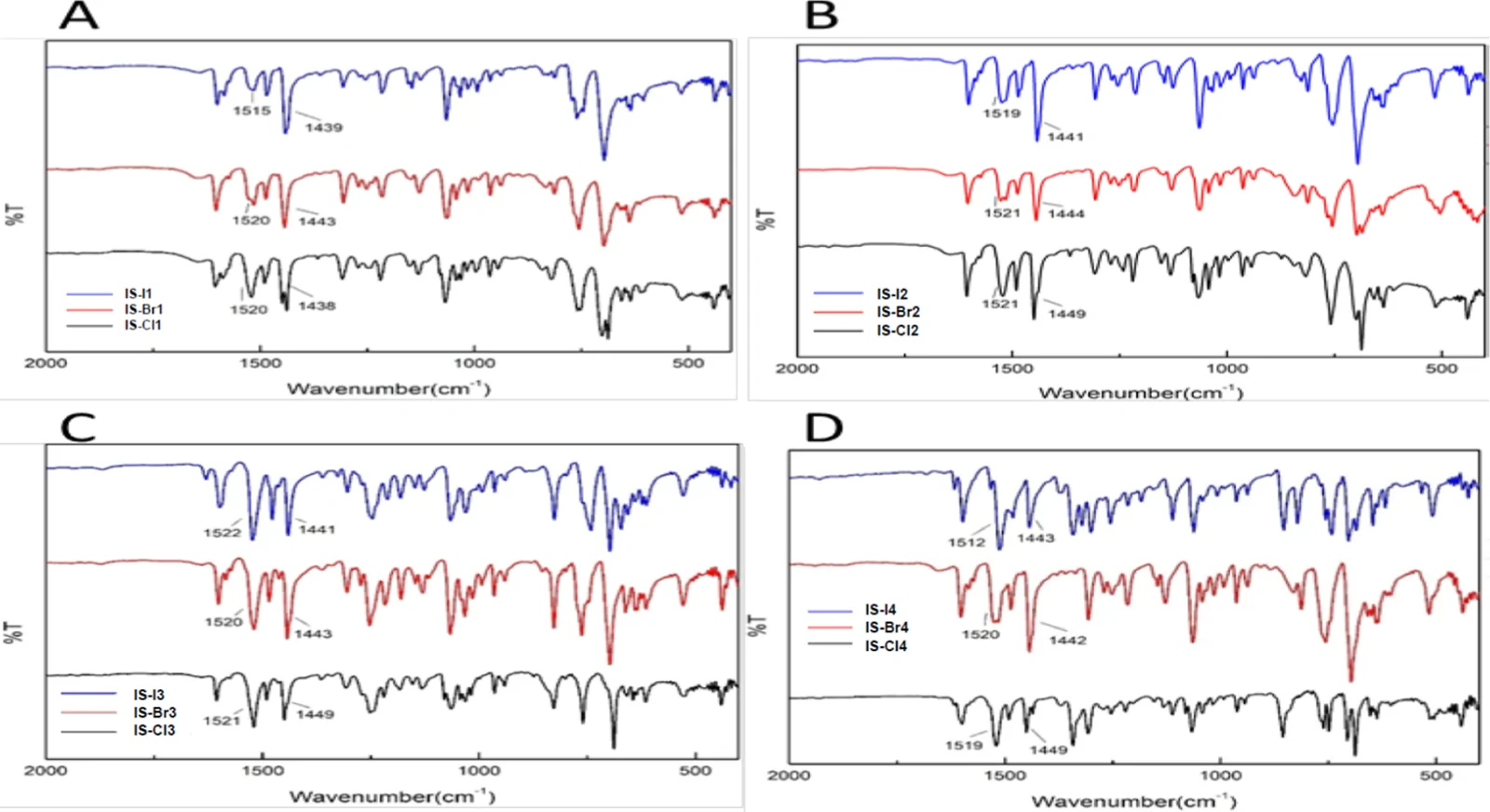
FT-IR spectra of (A) imidazolium salt of H-substituent groups on the aromatic group; (B) imidazolium salt of CH_3_-substituent groups on the aromatic group; (C) imidazolium salt of OCH_3_-substituent groups on the aromatic group; and (D) imidazolium salt of NO_2_-substituent groups on the aromatic group.

We first tested the reaction of benzyl azide **1a** and phenylacetylene **2** in the 3.0 mol % of copper­(I) chloride (CuCl), copper­(I) bromide (CuBr), and copper­(I) iodide (CuI) in commercial water at room temperature, where the mole ratio of benzyl azide **1a** to phenylacetylene **2** was 1.05. The reaction afforded the desired triazole product **3a** in 67%, 43%, and 37% yield, respectively (Table 1, entries 1–3). Replacement of CuCl with Cu­(I)­Cl_2_-based imidazolium salt **IS-Cl1**–**IS-Cl4** resulted in a reduced yield (Table 1, entries 4–7). Interestingly, when the CuBr was replaced with Cu­(I)­Br_2_-based imidazolium salt **IS-Br1**–**IS-Br4**, the reaction furnished a significant increase in yield, 72–98%, respectively (Table 1, entries 8–11). Meanwhile, the yield of **3a** seriously decreased when the CuI was replaced with Cu­(I)­I_2_-based imidazolium salt **IS-I1**–**IS-I4** (Table 1, entries 12–15). These observations might be rationalized as a result of an easier displacement of a bromo ligand from the copper center to initiate the catalytic cycle with the generation of copper acetylide. However, the catalyst metal–ligand is iodine which reacted with a lower yield. It is speculated the reason is that the atomic radius of iodine itself is larger than other halide atoms resulting in a higher steric hindrance, hence, it is difficult for copper to break from the product in the last step due to steric hindrance.^57–60^ Furthermore, the yield of product **3a** decreased while the amount of catalyst **IS-Br2** was changed (83% and 89%, entries 16, 17). To sum up, entry 9 corresponded to a slightly better yield than all other entries, so catalyst **IS-Br2** was chosen as the catalyst in the CuAAC reaction. Furthermore, the practical utility of this catalytic system was demonstrated by performing a gram-scale synthesis under the optimized conditions (Entry 9), which afforded the same excellent yield. This performance confirms that the developed methodology is highly effective for larger scale applications.

**Table 1:**
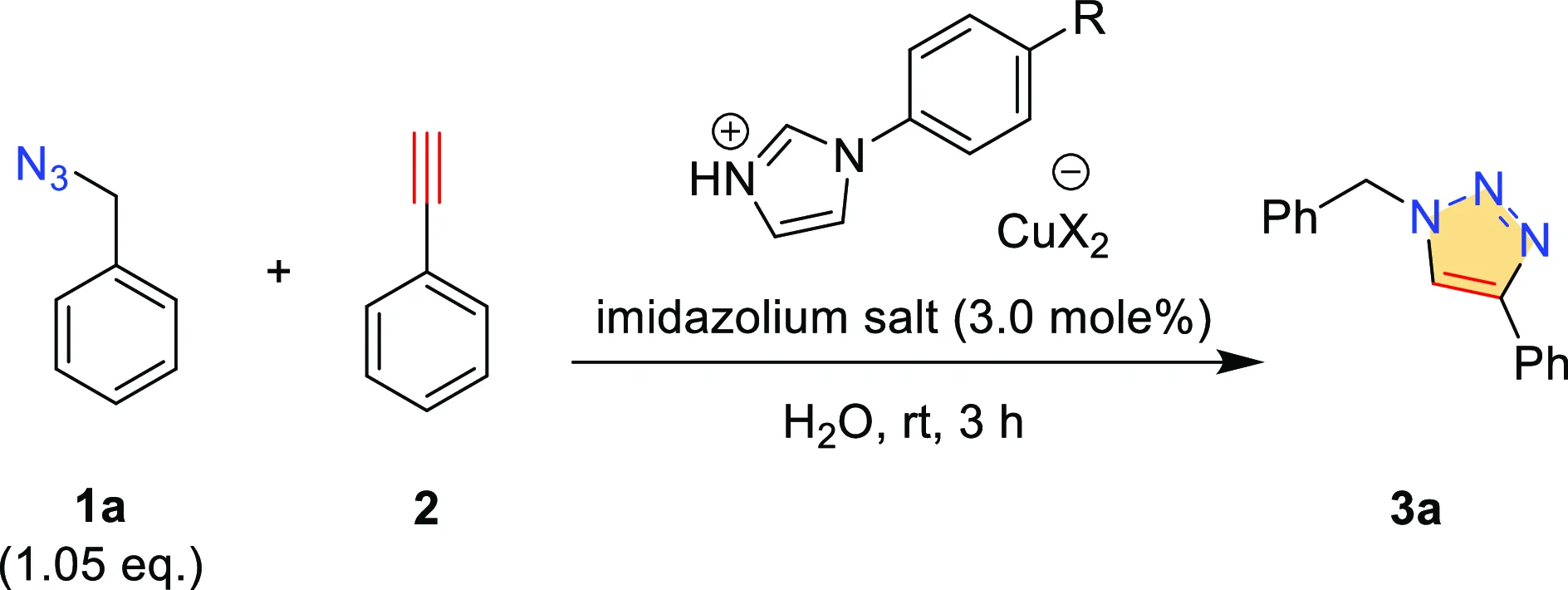
Screening of the Reaction Conditions

	catalyst (mole %)			
entry	cat	R	X	yield^a^
1	CuCl (3.0)			67%
2	CuBr (3.0)			43%
3	CuI (3.0)			37%
4	**IS-Cl1** (3.0)	H	Cl	60%
5	**IS-Cl2** (3.0)	CH_3_	Cl	21%
6	**IS-Cl3** (3.0)	OMe	Cl	56%
7	**IS-Cl4** (3.0)	NO_2_	Cl	45%
8	IS-Br1 (3.0)	H	Br	72%
9^b^	**IS-Br2** (3.0)	CH_3_	Br	98%
10	IS-Br3 (3.0)	OMe	Br	73%
11	IS-Br4 (3.0)	NO_2_	Br	87%
12	IS-I1 (3.0)	H	I	12%
13	IS-I2 (3.0)	CH_3_	I	27%
14	IS-I3 (3.0)	OMe	I	14%
15	IS-I4 (3.0)	NO_2_	I	15%
16	IS-Br2 (1.0)	CH_3_	Br	83%
17	IS-Br2 (5.0)	CH_3_	Br	89%

a Isolated yield.

b Gram-scale synthesis afforded the same yield.

Further investigation, including evaluating the reusability and stability of catalyst **IS-Br2** for the CuAAC reaction of benzyl azide **1a** with phenylacetylene **2**, was conducted under the optimum conditions obtained by the test as mentioned above (at room temperature, a reaction time of 3 h, catalyst **IS-Br2** amount 3.0 mol %, and a molar ratio of benzyl azide **1a** to phenylacetylene **2** of 1.05:1) that is based on the previous study of aryl imidazolium ionic liquid. **IS-Br2** could be easily separated from the reaction mixture by centrifugation with drying under a vacuum after washing with EtOAc 2–3 times. To ensure that the observed catalytic performance was not biased by mass loss during the process, the recovered catalyst was carefully weighed and resupplemented to the initial catalytic load (3.0 mol %) before each cycle. Using this process, three consecutive CuAAC procedures were conducted, for which the results are summarized in Table 2. Despite the precise resupplementation of the catalyst amount, a gradual decrease in product yield was observed, suggesting that the decline in reaction outcome is primarily attributed to the reduction in the intrinsic catalytic activity of **IS-Br2**, possibly due to structural degradation, poisoning, or active site leaching during the reaction cycles. To fully evaluate the practical appropriateness of this catalytic system for recycling applications, the nature of the handling process must be addressed. Because the reaction runs under a very low initial catalyst loading (3.0 mol %), the absolute mass of the recovered solid in our bench-scale setup is extremely small. The trace resupplementation of the catalyst before each run was a necessary measure to compensate for the unavoidable mechanical transfer losses caused by solid adhesion to centrifuge tubes and pipettes during the multistep washing sequence. Remarkably, despite these physical handling constraints, the catalytic system maintained excellent efficiency throughout the first three consecutive runs (98%, 94%, and 93% yield), with the performance only declining to 86% by the fourth cycle. This outcome underscores the excellent intrinsic chemical stability and robustness of the IS-Br2 matrix. Furthermore, such operational mass losses are highly scale-dependent and are significantly minimized upon scaling up (as demonstrated by the successful gram-scale synthesis), confirming that this system is highly appropriate for scalable and sustainable green chemistry applications.

**Table 2:**
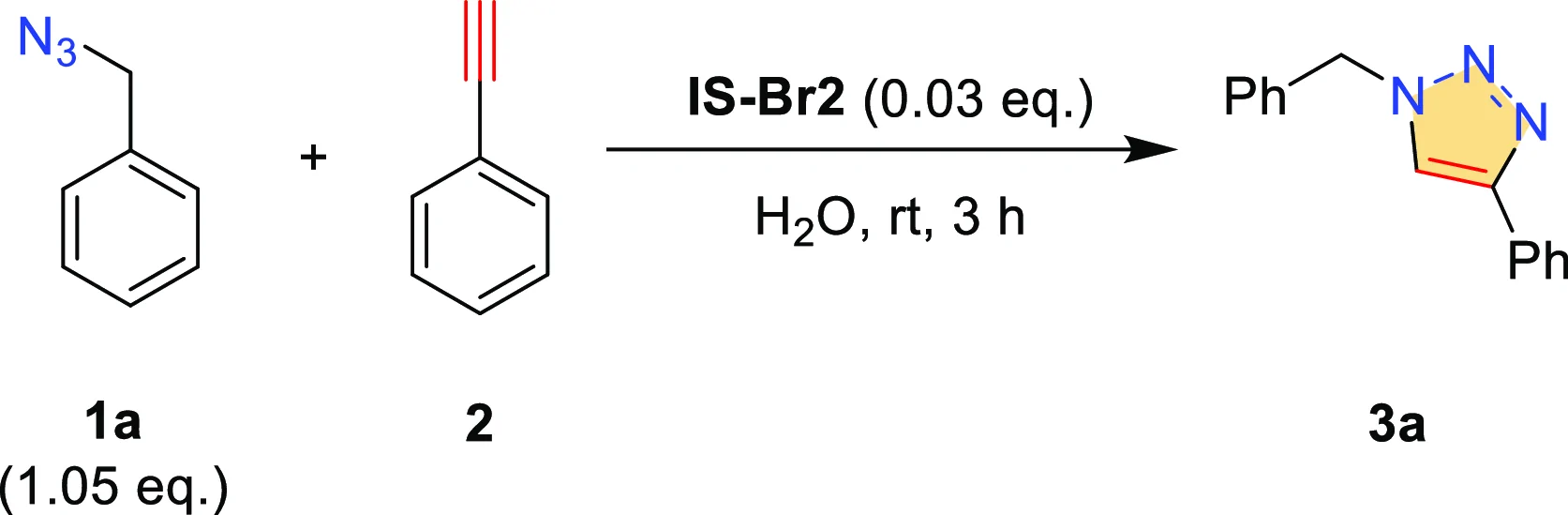

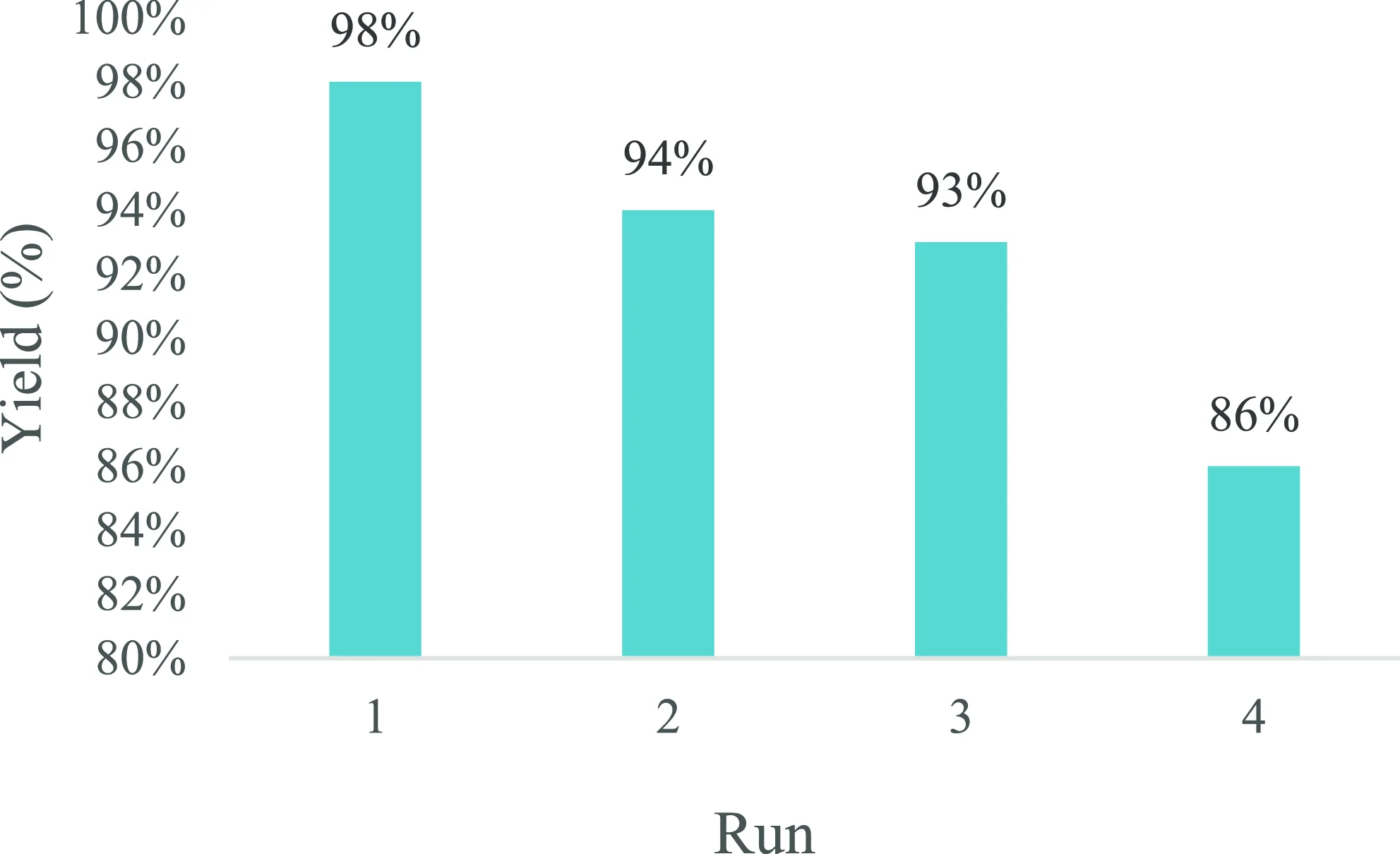
Recovery and reuse of Catalytic DATAIS System for CuAAC

With the best reaction conditions in optimization experiments (Table 1, entry 9), we extended the investigation to various substrate scopes (Scheme 3). Substrates **1b**–**1e** bearing para halogen-substituted benzyl groups were all suitable for this reaction and gave the corresponding products **3b**–**3e** in good to excellent yields. However, the responses of ortho-substituted benzyl substrate **3f** delivered the desired product in a relatively lower yield (75%) due to steric hindrance. In contrast, electron-withdrawing substituted of ester- and fully fluoride-benzyl substrates (**1g**-**1h**) and 2-naphthylmethyl (Nap)-substituted substrates **1i** proceeded with lower but still acceptable yields (46–67%) to the corresponding triazoles **3g**–**3i**. The reason is that the electron-withdrawing-substituted substrates reduce the coupling effectivity of the azide with copper during the reaction, resulting in poor reactivity. We attempted the reactions of hydrophobic substrates **1j**–**1l**. In the case of phenyl-substituted substrates **1j** and substrates **1l** bearing long-chain alkyl substitution, the desired products were reacted in 90% and 82%, respectively. On the other hand, the reaction of **1k** afforded **3k** with 40% isolated yield. It is noteworthy that several specific substrates, such as **3i** (46%) and **3k** (40%), afforded lower isolated yields compared to other derivatives. This decrease in isolation efficiency is directly attributed to the poor chemical reactivity of the starting materials **1i** and **1k** under the standard reaction conditions. Due to the less favorable electronic or steric effects of these specific substrates, the low catalyst loading of 0.03 eq. proved to be insufficient to drive the click reaction to completion within the designated time frame. As a result, an excessive amount of unreacted starting materials remained in the reaction mixture, directly leading to the lower isolated yields. This limitation suggests that a higher catalyst loading or extended reaction time might be required for these less reactive substrate variants.

**Scheme 3:**
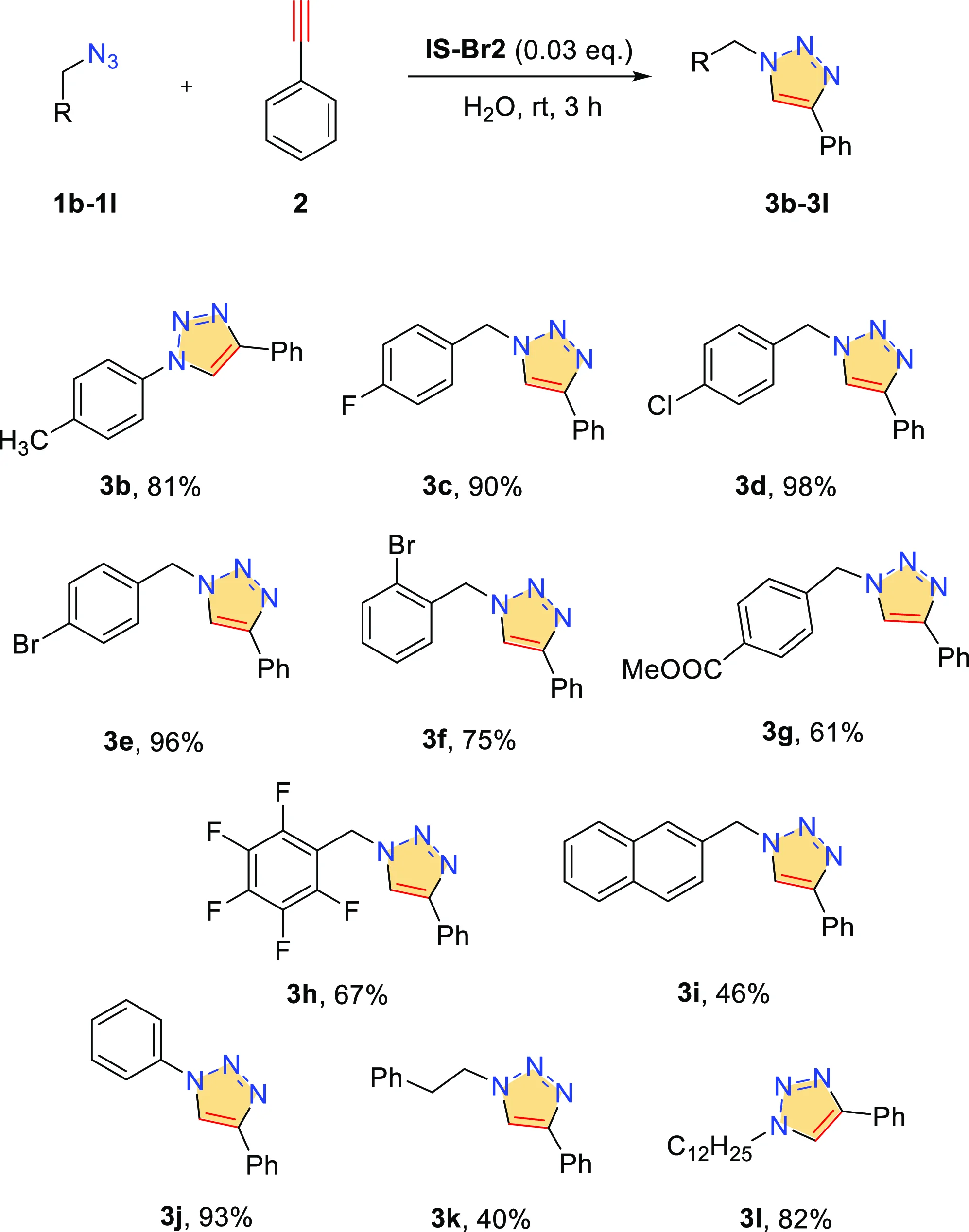
Substrate Scope

The proposed mechanism (Scheme 4 ^61–65^ ) fits perfectly with the heterogeneous and “on-water” nature of this catalytic system. Since the organic azide, terminal alkyne, and the aryl imidazolium matrix are hydrophobic, they are forced to aggregate at the aqueous interface due to the hydrophobic effect. This interfacial aggregation drastically increases the local concentration of reactants. The terminal alkyne reacts with the accessible [CuX2]^−^ active sites on the partially dissolved or suspended catalyst surface to generate Complex **A**. Subsequently, the organic azide easily accesses the localized copper center to form Complex **C**. The water molecules at the interface further stabilize the polar intermediates (Complexes **A**–**E**) via hydrogen bonding, promoting smooth oxidative coupling, ring contraction, and final product dissociation. This heterogeneous pathway not only drives high catalytic efficiency at room temperature but also enables convenient catalyst recovery.

**Scheme 4:**
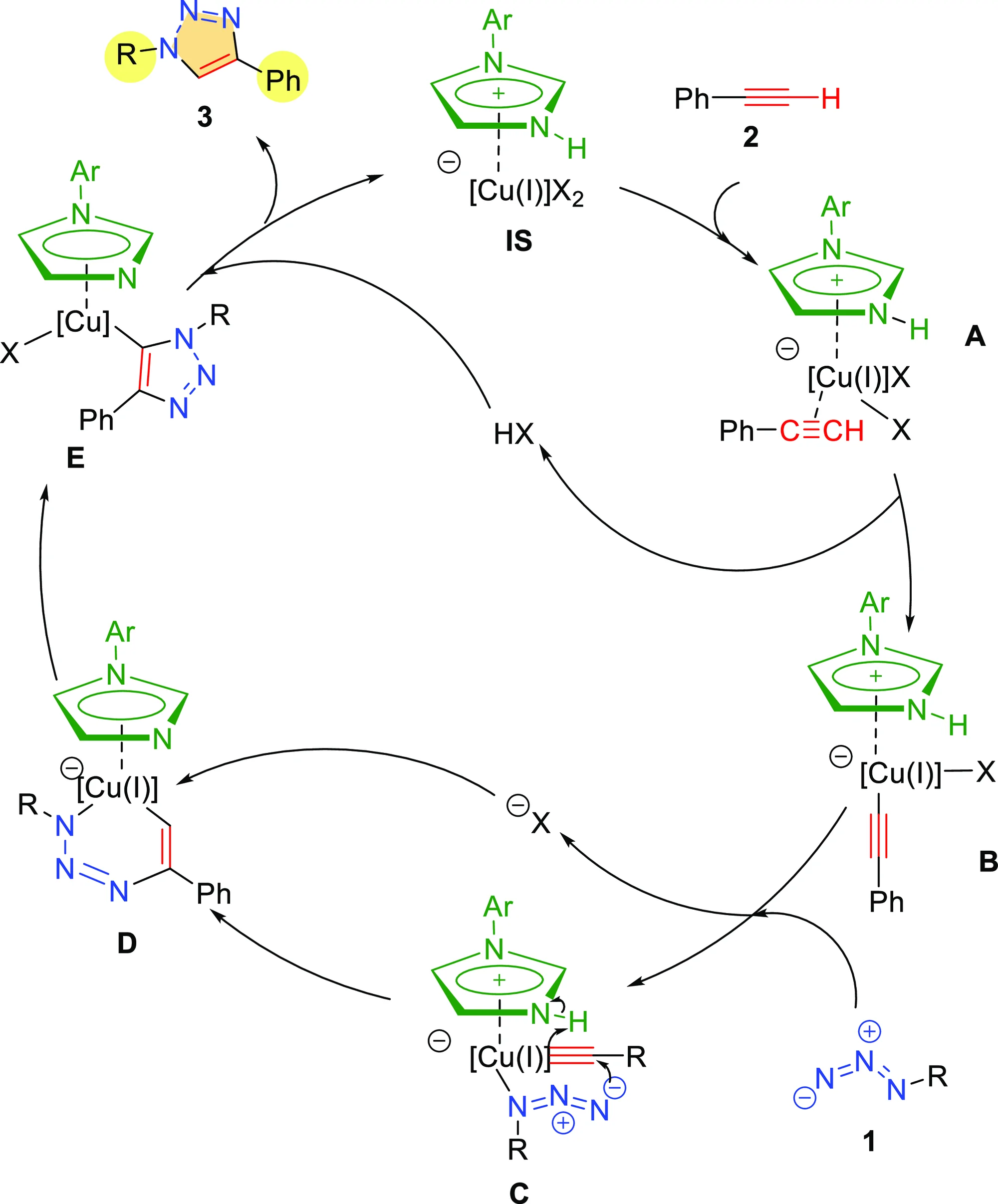
DATAIS-Catalyzed Mechanism of Azide–Alkyne Cycloaddition

## Conclusion

The use of CuAAC click chemistry is increasing tremendously daily, such as in organic synthesis, drug designation, material science, and polymer application. We herein report a novel imidazolium salt as the catalyst, which was designed based on a tunable aryl imidazolium moiety with dual Brønsted and Lewis acidic sites (B-L MISs). These B-L MISs exhibited good reactivity for synthetic 1,4-disubstituted 1,2,3-triazoles by CuAAc reaction. The catalyst showed high atom economy and high thermal stability, besides the imidazolium salt could be recovered and reused for click chemistry. This provides more possibilities for the industrial process application of CuAAc reaction, which provides another eco-friendly method with the potential for scaling up in the field of CuAAC.

## Experiments

### General Information

Some reactions were performed in dry glassware and others in Ace pressure tubes. Dichloromethane, tetrahydrofuran, toluene, and acetonitrile are purified through a safety purification system containing activated Al_2_O_3_ and dried. All reagents obtained from commercial sources were used without further purification. Column chromatography was performed on silica 60 (23–60 mesh, E. Merck) or Silica 60A (15–40 μm, E. Merck) TLC was performed on silica gel 60 F254 (0.25 mm, E. Merck) precoated glass plates; transmission spray Ce­(NH_4_)_2_(NO_3_)_6_ (0.5 g), (NH_4_)_6_Mo_7_O_24_ (24g), and H_2_SO_4_ (28 mL) in water (500 mL), followed by heating on a hot plate for detection. Detection of aromatic compounds was achieved via 254 nm UV radiation on TLC using the UVGL-25 compact UV lamp. Melting points were determined on a Mel-Temp II (Laboratory Equipment). ^1^H and ^13^C NMR spectra were recorded with a 400 MHz instrument. Chemical shifts are in ppm and are generated by a CDCl_3_ of 7.26 or a DMSO lock signal of 2.49. Infrared spectra were obtained by an FT-IR spectrometer using ATR. Mass spectra were analyzed on a Finnigan LTQ-OrbitrapxL instrument equipped with ESI, APCI, EI, or FAB sources.

### Synthesis and Characterization of Aryl Imidazolium Salts

#### General Procedure and for Preparation of **IS-Cl1–IS-I4**

To a solution of 3-phenyl-1*H*-imidazolium chloride **B** (100 mg, 1.0 equiv), copper­(I) halide (1.2 equiv) was added in ethanol (5 mL). The reaction mixture was stirred at room temperature for 24 h. The solvent was removed under reduced pressure, and the reaction mixture was washed with ethanol several times. The solvent was dried to give the desired DATAIS **IS-Cl1**–**IS-I4**.

#### 3-Phenyl-1*H*-Imidazolium Dichlorocuprate­(I) (**IS-Cl1**)

Prepared according to the general procedure; white gray solid; yield: 112.2 mg (73%); mp 234–238 °C; FTIR ν 1606, 1523, 1438, 1069, 759, 686 cm^–1^; EA 39.21% (C), 3.26% (H), 9.49% (N); HRMS (ESI, [M]^+^) calcd for C_9_H_9_N_2_ 145.07657, found 145.07651; HRMS (ESI, [M]^−^) calcd for Cl_2_Cu 132.8673; found, 132.8676.

#### 3-(4-Methylphenyl)-1*H*-imidazolium Dichlorocuprate­(I) (**IS-Cl2**)

Prepared according to the general procedure; yellow solid; yield: 90.4 mg (60%); mp 225–234 °C; FTIR ν 1606, 1516, 1447, 1066, 759, 686 cm^–1^; EA 36.43% (C), 3.06% (H), 8.77% (N); HRMS (ESI, [M]^+^) calcd for C_10_H_11_N_2_ 159.09222; found, 159.09203; HRMS (ESI, [M]^−^) calcd for Cl_2_Cu 132.8673; found, 132.8676.

#### 3-(4-Methoxyphenyl)-1*H*-imidazolium Dichlorocuprate­(I) (**IS-Cl3**)

Prepared according to the general procedure; yellow solid; yield: 103.7 mg (71%); mp 233–236 °C; FTIR ν 1606, 1521, 1447, 1251, 1062, 825, 71, 688 cm^–1^; EA 36.5% (C), 3.13% (H), 12.28% (N); HRMS (ESI, [M]^+^) calcd for C_10_H_11_N_2_O 175.08714; found, 175.08717; HRMS (ESI, [M]^−^) calcd for Cl_2_Cu 132.8673; found, 132.8704.

#### 3-(4-Nitrophenyl)-1*H*-imidazolium Dichlorocuprate­(I) (**IS-Cl4**)

Prepared according to the general procedure; yellow solid; yield: 118 mg (83%); mp 255–262 °C; FTIR ν 1601, 1521, 1447, 1343, 1305, 1066, 851, 688 cm^–1^; EA 32.19% (C), 2.27% (H), 12.28% (N); HRMS (ESI, [M]^+^) calcd for C_9_H_8_N_3_O_2_ 190.06165; found, 190.06057; HRMS (ESI, [M]^−^) calcd for Cl_2_Cu 132.8673; found, 132.8678.

#### 3-Phenyl-1*H*-Imidazolium Dibromocuprate­(I) (**IS-Br1**)

Prepared according to the general procedure; brown solid; yield: 45 mg (22%); mp 184–188 °C; FTIR ν 1603, 1445, 1308, 1064, 759, 695 cm^–1^; EA 31.07% (C), 2.60% (H), 7.48% (N); HRMS (ESI, [M]^+^) calcd for C_9_H_9_N_2_ 145.0766; found, 145.0764; HRMS (ESI, [M]^−^) calcd for Br_2_Cu 220.7663; found, 220.7661.

#### 3-(4-Methylphenyl)-1*H*-imidazolium Dibromocuprate­(I) (**IS-Br2**)

Prepared according to the general procedure; brown solid; yield: 53 mg (33%); mp 214–217 °C; FTIR ν 1601, 1528, 1442, 1308, 1069, 754, 697 cm^–1^; EA 30.88% (C), 2.64% (H), 7.14% (N); HRMS (ESI, [M]^+^) calcd for C_10_H_11_N_2_ 159.0922; found, 159.0923; HRMS (ESI, [M]^−^) calcd for Br_2_Cu 220.7663; found, 220.7661.

#### 3-(4-Methoxyphenyl)-1*H*-imidazolium Dibromocuprate­(I) (**IS-Br3**)

Prepared according to the general procedure; brown solid; yield: 100.8 mg (65%); mp 165–170 °C; FTIR ν 1606, 1516, 1440, 1253, 1066, 1028, 825, 693 cm^–1^; EA 32.59% (C), 2.78% (H), 7.50% (N); HRMS (ESI, [M]^+^) calcd for C_10_H_11_N_2_O 175.0871; found, 175.0871; HRMS (ESI, [M]^−^) calcd for Br_2_Cu 220.7663; found, 220.7664.

#### 3-(4-Nitrophenyl)-1*H*-imidazolium Dichlorocuprate­(I) (**IS-Br4**)

Prepared according to the general procedure; brown solid; yield: 116.2 mg (76%); mp 162–167 °C; FTIR ν 1603, 1518, 1442, 1305, 1059, 754, 697 cm^–1^; EA 27.34% (C), 2.34% (H), 6.51% (N); HRMS (ESI, [M]^+^) calcd for C_9_H_8_N_3_O_2_ 190.0617, found 190.0604; HRMS (ESI, [M]^−^) calcd for Br_2_Cu 220.7663; found, 220.7660.

#### 3-Phenyl-1*H*-Imidazolium Diiodocuprate­(I) (**IS-I1**)

Prepared according to the general procedure; brown solid; yield: 112.6 mg (66%); mp 259–263 °C; FTIR ν 1599, 1485, 1445, 1064, 756, 695 cm^–1^; EA 23.62% (C), 1.77% (H), 5.45% (N); HRMS (ESI, [M]^+^) calcd for C_9_H_9_N_2_ 145.0766; found, 145.0767; HRMS (ESI, [M]^−^) calcd for I_2_Cu 316.7385; found, 316.7393.

#### 3-(4-Methylphenyl)-1*H*-imidazolium Diiodocuprate­(I) (**IS-I2**)

Prepared according to the general procedure; brown solid; yield: 99.4 mg (60%); mp 120–128 °C; FTIR ν 1603, 1525, 1440, 1308, 1062, 752, 695 cm^–1^; EA 21.52% (C), 1.92% (H), 5.13% (N); HRMS (ESI, [M]^+^) calcd for C_10_H_11_N_2_ 159.0922; found, 159.0922; HRMS (ESI, [M]^−^) calcd for I_2_Cu 316.7385; found, 316.7398.

#### 3-(4-Methoxyphenyl)-1*H*-imidazolium Diiodocuprate­(I) (**IS-I3**)

Prepared according to the general procedure; brown solid; yield: 127.6 mg (79%); mp 234–242 °C; FTIR ν 1601, 1523, 1442, 1248, 1064, 825, 740, 695 cm^–1^; EA 23.96% (C), 2.05% (H), 5.26% (N); HRMS (ESI, [M]^+^) calcd for C_10_H_11_N_2_O 175.0866; found, 175.0866; HRMS (ESI, [M]^−^) calcd for I_2_Cu 316.7385; found, 316.7199.

#### 3-(4-Nitrophenyl)-1*H*-imidazolium Diiodocuprate­(I) (**IS-I4**)

Prepared according to the general procedure; brown solid; yield: 130.4 mg (80%); mp 233–239 °C; FTIR ν 1599, 1511, 1440, 1343, 1298, 1109, 1062, 851, 742, 702 cm^–1^; EA 20.79% (C), 1.54% (H), 7.69% (N); HRMS (ESI, [M]^+^) calcd for C_9_H_8_N_3_O_2_ 190.0617; found, 190.0609; HRMS (ESI, [M]^−^) calcd for I_2_Cu 316.7385; found, 316.7392.

### Procedure and Characterization of CuAAC Products

#### General Procedure for CuAAC Reaction

In a vial containing a magnetic stir bar were added **IS-Br2** (1.0 mol % for **1b**–**1c** and **1l**; 3.0 mol % for **1a, 1d–1f**, and **1h**–**1k**; 0.5 mol % for **1g**), water (solvent, 1.0 mL per 100 mg of **1**), followed by phenylacetylene **2** (1.0 equiv) and azide compounds **1a**–**1l** (1.05 equiv). The reaction mixture was stirred for 3 h at room temperature. The mixture was quenched with an ammonia solution and diluted with EtOAc. After the mixture was extracted with brine. The organic layer was dried over MgSO_4_, then filtered, and concentrated with reduced pressure to remove the solvent. Afterward, the residue was purified by column chromatography (eluent: ethyl acetate/*n*-hexane) to afford the desired 1,2,3-triazole products **3a**-**3l**.

#### Gram-Scale Synthesis of 1,2,3-Triazole **3a**

In a vial containing a magnetic stirrer bar were added **IS-Br2** (3.0 mol %), water (solvent, 10 mL), followed by phenylacetylene **2** (0.73 g, 7.15 mmol, 1.0 equiv) and azide compounds **1a** (1.0 g, 7.51 mmol, 1.05 equiv). The reaction mixture was stirred for 3 h at room temperature. The mixture was quenched with an ammonia solution and diluted with EtOAc. After that, the mixture was extracted with brine. The organic layer was dried over MgSO_4_, then filtered, and concentrated with reduced pressure to remove the solvent. Afterward, the residue was purified by column chromatography (eluent: ethyl acetate/*n*-hexane = 5/95) to afford the desired 1,2,3-triazole product **3a** as a white solid (2.3 g, 98% isolated yield).

#### 1-Benzyl-4-phenyl-1*H*-1,2,3-triazole (**3a**)

Prepared according to the general procedure discussed above; white solid; yield:164 mg (98%); *R*_*f*_ 0.50 (EtOAc/*n*-Hexane = 1/2); mp 125–130 °C; FTIR ν 3141, 3083, 3029, 2977, 1985, 1966, 1606, 1581, 1553, 1495, 1483, 1468, 1451, 1428, 1361, 1333, 1322, 1294, 1283, 1255, 1223, 1208, 1194, 1175, 1159, 1140, 1075, 1047, 1026, 1002, 972, 952, 858, 826, 808, 767, 726, 693, 616, 588, 507, 480 cm^–1^; ^1^H NMR (400 MHz, CDCl_3_): δ 7.80 (dd, *J* = 8.3, 1.2 Hz, 2H), 7.66 (d, *J* = 1.9 Hz, 1H), 7.45–7.24 (m, 8H), 5.58 (s, 2H); ^13^C­{^1^H} NMR (101 MHz, CDCl_3_): δ 148.2, 134.6, 130.5, 129.1, 128.8, 128.1, 128.0, 125.6, 119.5, 94.1, 54.2; HRMS (ESI, [M + Na]^+^) calcd for C_15_H_13_N_3_Na 258.1007; found, 258.1009.

#### 4-Phenyl-1-(*p*-tolyl)-1*H*-1,2,3-triazole (3b)

Prepared according to the general procedure discussed above; white solid; yield: 135 mg (81%); *R*_*f*_ 0.70 (EtOAc/Hex = 1/2); mp 171–175 °C; FTIR ν 1519, 1454, 814, 762, 690 cm^–1^; ^1^H NMR (400 MHz, CDCl_3_): δ 8.16 (s, 1H), 7.94–7.89 (m, 2H), 7.70–7.64 (m, 2H), 7.46 (t, *J* = 7.5 Hz, 2H), 7.40–7.32 (m, 3H), 2.44 (s, 3H).; ^13^C­{^1^H} NMR (101 MHz, CDCl_3_): δ 148.3, 138.9, 134.8, 130.3, 130.3, 128.9, 128.4, 125.8, 120.4, 117.6, 21.1; HRMS (ESI, [M + H]^+^) calcd for C_15_H_14_N_3_ 236.1188; found, 236.1183.

#### 1-(4-Fluorobenzyl)-4-phenyl-1*H*-1,2,3-triazole (**3c**)

Prepared according to the general procedure discussed above; white solid; yield: 143 mg (90%); *R*_*f*_ 0.50 (EtOAc/Hex = 1/2); mp 128–131 °C; FTIR ν 3095, 2923, 2853, 1603, 1510, 1463, 1352, 1225, 1075, 765, 696 cm^–1^; ^1^H NMR (400 MHz, CDCl_3_): δ 7.80 (d, *J* = 7.1 Hz, 2H), 7.66 (s, 1H), 7.40 (t, *J* = 7.5 Hz, 2H), 7.35–7.27 (m, 3H), 7.08 (t, *J* = 8.6 Hz, 2H), 5.55 (s, 2H); ^13^C­{^1^H} NMR (101 MHz, CDCl_3_): δ 162.9, 148.3, 130.5, 130.4, 129.9, 128.8, 128.2, 125.7, 119.3, 116.2, 53.5; HRMS (ESI, [M + H]^+^) calcd for C_15_H_13_FN_3_ 254.1094; found, 254.1096.

#### 1-(4-Chlorobenzyl)-4-phenyl-1*H*-1,2,3-triazole (**3d**)

Prepared according to the general procedure discussed above; white solid; yield: 264 mg (98%); *R*_*f*_ 0.50 (EtOAc/Hex = 1/2); mp 143–148 °C; FTIR ν 3085, 2978, 1685, 1597, 1492, 1463, 1412, 1352, 1221, 1093, 1015, 805, 762, 687 cm^–1^; ^1^H NMR (400 MHz, CDCl_3_): δ 7.79 (d, *J* = 7.5 Hz, 2H), 7.67 (s, 1H), 7.43–7.28 (m, 5H), 7.24–7.22 (m, 2H), 5.53 (s, 2H); ^13^C­{^1^H} NMR (101 MHz, CDCl_3_): δ 148.2, 134.9, 133.0, 130.1, 129.4 (2C), 128.8, 128.4, 125.8, 119.5, 53.6; HRMS (ESI, [M – H]^−^) calcd for C_15_H_11_ClN_3_ 268.0642; found, 268.0642.

#### 1-(4-Bromobenzyl)-4-phenyl-1*H*-1,2,3-triazole (**3e**)

Prepared according to the general procedure discussed above; white solid; yield: 302 mg (96%); *R*_*f*_ 0.50 (EtOAc/Hex = 1/2); mp 151–155 °C; FTIR ν 3126, 3085, 2924, 1590, 1480, 1217, 1074, 1013, 806, 767, 696, 492 cm^–1^; ^1^H NMR (400 MHz, CDCl_3_): δ 7.81–7.75 (m, 2H), 7.67 (s, 1H), 7.53–7.47 (m, 2H), 7.42–7.35 (m, 2H), 7.34–7.28 (m, 1H), 7.20–7.14 (m, 2H), 5.51 (s, 2H); ^13^C­{^1^H} NMR (101 MHz, CDCl_3_): δ 133.5, 132.3, 130.1, 129.7, 128.8, 128.4, 128.1, 125.7, 123.0, 119.5, 53.6; HRMS (ESI, [M – H + 2Na]^−^) calcd for C_15_H_11_BrN_3_Na_2_ 357.9932; found, 357.9932.

#### 1-(2-Bromobenzyl)-4-phenyl-1*H*-1,2,3-triazole (**3f**)

Prepared according to the general procedure discussed above; white solid; yield: 236 mg (75%); *R*_*f*_ 0.50 (EtOAc/Hex = 1/2); mp 141–146 °C; FTIR ν 3083, 3065, 2921, 1482, 1463, 1441, 1429, 1221, 1193, 1181, 1079, 1049, 1034, 976, 912, 820, 762, 687 cm^–1^; ^1^H NMR (400 MHz, CDCl_3_): δ 7.82 (d, *J* = 7.3 Hz, 2H), 7.74 (s, 1H), 7.45–7.31 (m, 5H), 7.27 (d, *J* = 6.9 Hz, 2H), 5.57 (s, 2H); ^13^C­{^1^H} NMR (101 MHz, CDCl_3_): δ 148.2, 134.8, 133.1, 130.2 (2C), 129.3, 129.3, 128.8, 128.3 (2C), 125.7, 119.5, 53.5; HRMS (ESI, [M]^+^) calcd for C_15_H_12_BrN_3_ 314.0248; found, 314.0319.

##### Methyl-4-((4-phenyl-1*H*-1,2,3-triazol-1-yl)­methyl)­benzoate (**3g**)

Prepared according to the general procedure discussed above; white solid; yield: 89 mg (61%); *R*_*f*_ 0.76 (EtOAc/Hex = 1/1); mp 161–167 °C; FTIR ν 3083, 3059, 2954, 1712, 1613, 1485, 1465, 1441, 1418, 1356, 1317, 1279, 1224, 1194, 1112, 1078, 1048, 1021, 838, 800, 770, 744, 713, 695 cm^–1^; ^1^H NMR (400 MHz, CDCl_3_): δ 8.05 (d, *J* = 8.3 Hz, 2H), 7.81 (d, *J* = 7.1 Hz, 2H), 7.69 (s, 1H), 7.34–7.43 (m, 5H), 5.65 (s, 2H), 3.92 (s, 3H). ^13^C­{^1^H} NMR (101 MHz, CDCl_3_): δ 166.4, 148.5, 139.5, 130.6, 130.4, 130.3, 128.8, 128.3, 127.9, 127.8, 125.7, 119.6, 53.7, 52.3; HRMS (ESI, [M + H]^+^) calcd for C_17_H_16_N_3_O_2_ 294.1242; found, 294.1247.

#### 1-[(Perfluorophenyl)­methyl]-4-phenyl-1*H*-1,2,3-triazole (**3h**)

Prepared according to the general procedure discussed above; white solid; yield: 218 mg (67%); *R*_*f*_ 0.73 (EtOAc/Hex = 1/1); mp 161–166 °C; FTIR ν 3124, 2969, 2924, 2846, 1658, 1521, 1503, 1464, 1424, 1226, 1196, 1163, 1129, 1081, 1021, 968, 918, 818, 764, 692, 652, 507, 453 cm^–1^; ^1^H NMR (400 MHz, CDCl_3_): δ 7.85–7.76 (m, 3H), 7.41 (m, 2H), 7.37–7.30 (m, 1H), 5.67 (t, *J* = 1.7 Hz, 2H); ^13^C­{^1^H} NMR (101 MHz, CDCl_3_): δ 148.4, 130.0, 128.8 (2C), 128.4 (2C), 125.8 (2C), 119.6, 94.0, 40.9; HRMS (ESI, [M – H]^−^) calcd for C_15_H_8_F_5_N_3_ 324.0560; found, 324.0561.

#### 1-(Naphthalen-2-ylmethyl)-4-phenyl-1*H*-1,2,3-triazole (**3i**)

Prepared according to the general procedure discussed above; white solid; yield: 131 mg (46%); *R*_*f*_ 0.63 (EtOAc/Hex = 1/1); mp 133–137 °C; FTIR ν 3123, 3055, 2925, 1460, 1425, 1365, 1307, 1219, 1187, 1079, 1034, 969, 904, 864, 835, 816, 757, 693 cm^–1^; ^1^H NMR (400 MHz, CDCl_3_): δ 7.91–7.82 (m), 7.81–7.76 (m), 7.68 (s), 7.56–7.49 (m), 7.44–7.35 (m), 7.34–7.27 (m), 5.75 (s); ^13^C­{^1^H} NMR (101 MHz, CDCl_3_): δ 133.2, 129.2, 128.8 (2C), 128.3, 127.9 (2C), 127.8 (2C), 127.4, 126.8 (2C), 125.7 (2C), 125.3, 119.7, 54.6; HRMS (ESI, [M]^+^) calcd for C_19_H_15_N_3_Na 308.1163; found, 308.1160.

#### 1,4-Diphenyl-1*H*-1,2,3-triazole (**3j**)

Prepared according to the general procedure discussed above; white solid; yield: 165 mg (93%); *R*_*f*_ 0.65 (EtOAc/Hex = 1/1); mp 172–183 °C; FTIR ν 3427, 3121, 3098, 1597, 1503, 1466, 1414, 1228, 1039, 754, 688 cm^–1^; ^1^H NMR (400 MHz, CDCl_3_): δ 8.20 (s, 1H), 7.92 (d, *J* = 7.1 Hz, 2H), 7.80 (d, *J* = 7.5 Hz, 2H), 7.55 (t, *J* = 7.8 Hz, 2H), 7.47 (dd, *J* = 8.8, 6.2 Hz, 3H), 7.37 (t, *J* = 7.4 Hz, 1H); ^13^C­{^1^H} NMR (101 MHz, CDCl_3_): δ 148.4, 137.0, 130.2, 129.8, 128.8, 128.4, 125.8, 120.5, 117.6, 77.3, 77.0, 76.7; HRMS (ESI, [M + H]^+^) calcd for C_14_H_12_N_3_ 222.1031; found, 222.1032.

#### 1-Phenethyl-4-phenyl-1*H*-1,2,3-triazole (**3k**)

Prepared according to the general procedure discussed above; white solid; yield: 65 mg, (40%); *R*_*f*_ 0.76 (EtOAc/Hex = 1/1); mp 132–142 °C; FTIR ν 3084, 3062, 3028, 1455, 1223, 1085 cm^–1^; ^1^H NMR (400 MHz, CDCl_3_): δ 7.76 (d, *J* = 7.8 Hz, 2H), 7.47 (s, 1H), 7.41 (t, *J* = 7.5 Hz, 2H), 7.33–7.28 (m, 4H), 7.14 (d, *J* = 6.7 Hz, 2H), 4.63 (t, *J* = 7.2 Hz, 2H), 3.25 (t, *J* = 7.2 Hz, 2H); ^13^C­{^1^H} NMR (101 MHz, CDCl_3_): δ 147.4, 137.0, 130.6, 128.8, 128.8, 128.7, 128.0, 127.1, 125.6, 119.9, 77.3, 77.0, 76.7, 51.7, 36.8; HRMS (ESI, [M]^+^) calcd for C_16_H_15_N_3_ 250.1344; found, 250.1342.

#### 1-Dodecyl-4-phenyl-1*H*-1,2,3-triazole (**3l**)

Prepared according to the general procedure discussed above; white solid; yield: 126 mg (82%); *R*_*f*_ 0.74 (EtOAc/Hex = 1/1); mp 71–79 °C; FTIR ν 2916, 2847, 1464, 1216, 1199, 1184, 1078, 1053, 1025, 977, 913, 840, 761, 723, 694 cm^–1^; ^1^H NMR (400 MHz, CDCl_3_): δ 7.93–7.89 (m, 2H), 7.83 (s, 1H), 7.49–7.44 (m, 2H), 7.41–7.36 (m, 1H), 4.44 (t, *J* = 7.2 Hz, 2H), 2.03–1.93 (m, 2H), 1.26 (s, 18H), 0.88 (t, *J* = 6.8 Hz, 3H); ^13^C­{^1^H} NMR (101 MHz, CDCl_3_): δ 147.8, 130.8, 128.9, 128.2, 125.8, 119.4, 50.5, 32.0, 30.5, 29.7, 29.2, 26.8, 26.6, 22.8, 14.2; HRMS (ESI, [M + H]^+^) calcd for C_20_H_32_N_3_ 314.2518; found, 314.2605.

## Supplementary Material



## Data Availability

The data underlying this study are available in the published article and its Supporting Information.

## Notes

General experimental procedures, FTIR, ^1^H, ^13^C {^1^H} NMR spectra, and HRMS data for compounds **3a**–**3l** and IS IS-Cl1–IS-I4 (PDF)
