# Supplementary material for: Development of Noval, Recyclable, and Tunable Aryl Imidazolium Salts with a Dual Brønsted–Lewis Acid Character as Catalysts for the Copper(I)-Catalyzed Azide–Alkyne Cycloaddition Reaction (CuAAC)
Source: ACS Org Inorg Au. 2026 Aug 13;6(5):697–706. doi: 10.1021/acsorginorgau.6c00066 (PMC13650945; doi:10.1021/acsorginorgau.6c00066)
Supplement: Supplementary file 1 [file gg6c00066_si_001.pdf]

Supporting Information

Development of Noval, Recyclable, and Tunable Aryl Imidazolium Salts with Dual Brønsted–Lewis Acid Character as Catalysts for the Copper(I)-Catalyzed Azide-Alkyne Cycloaddition Reaction (CuAAC)

Wen-Yueh Ho,[a] Yu-Hsuan Yang,[b] Jyun-Siao Chen,[b] Ratnnadeep C. Sawant,[c] Tzu-Hsin Shu,[b] Pei-Yu Chen,[b] Chia-Yun Chang,[b] Shun-Yuan Luo[b*]

[a]Department of Food & Drug Industry and Inspective Technology, Chia Nan University of Pharmacy and Science, Tainan 717, Taiwan

[b]Department of Chemistry, National Chung Hsing University, Taichung 402, Taiwan

[c]Department of Chemistry, Dr. Ambedkar College, Deekshabhoomi, Nagpur - 440010, India

E-mail: *syluo@dragon.nchu.edu.tw*

**Contents**	**Page**
Physicochemical Characterization of Ionic Liquids	2-4
NMR spectrum of important compounds	5-28

Table S1. Solubility Data of the Synthesized Ionic Liquids (IL-Cl1 to IL-I4) in Various Organic Solvents.[1]

	DMSO	DMF	Acetone	DCM	THF	EA	Hex
ε [a]	46.7	36.7	20.7	8.93	7.58	6.02	1.88
**IL-Cl1**	+	+	±	±	±	—	—
**IL-Cl2**	+	+	±	±	±	—	—
**IL-Cl3**	+	+	±	±	±	—	—
**IL-Cl4**	+	+	±	±	±	—	—
**IL-Br1**	+	+	±	±	±	—	—
**IL-Br2**	+	+	±	±	±	—	—
**IL-Br3**	+	+	±	±	±	—	—
**IL-Br4**	+	+	±	±	±	—	—
**IL-I1**	+	+	±	±	±	±	—
**IL-I2**	+	+	±	±	±	±	—
**IL-I3**	+	+	±	±	±	±	—
**IL-I4**	+	+	±	±	±	±	—

[a] Dielectric constant (ε); [b]Solubility symbols: + = soluble (> 1 mg/mL); ± = sparingly soluble (1 mg/mL, fully soluble upon heating/agitation); - = insoluble (< 1 mg/mL) and all measurements were conducted at 25 °C.

The solubility of the synthesized ionic liquids was evaluated across various organic solvents with different dielectric constants (ε). As shown in Table S1, a distinct correlation is observed: the solubility of the ionic liquids increases significantly in solvents with higher dielectric constants. This behavior is consistent with the intrinsic ionic nature of the synthesized compounds, as the charge-separated species are more effectively stabilized by high-polarity solvents via ion-dipole interactions. This solvent-dependent solubility profile further confirms the ionic character and structural design of the synthesized ILs, aligning with their classification as ionic liquids.

Table S2. TGA-derived decomposition temperatures (T_d) of aryl imidazolium ionic liquids with different cation substituents.

Entry	ILs	$T_{d,5\%}$ (℃)	$T_{d,60\%}$ (℃)	$T_{d,90\%}$ (℃)	Anion	Figure
1	IL-Cl1	188	236	411	$[CuCl_2^-]$	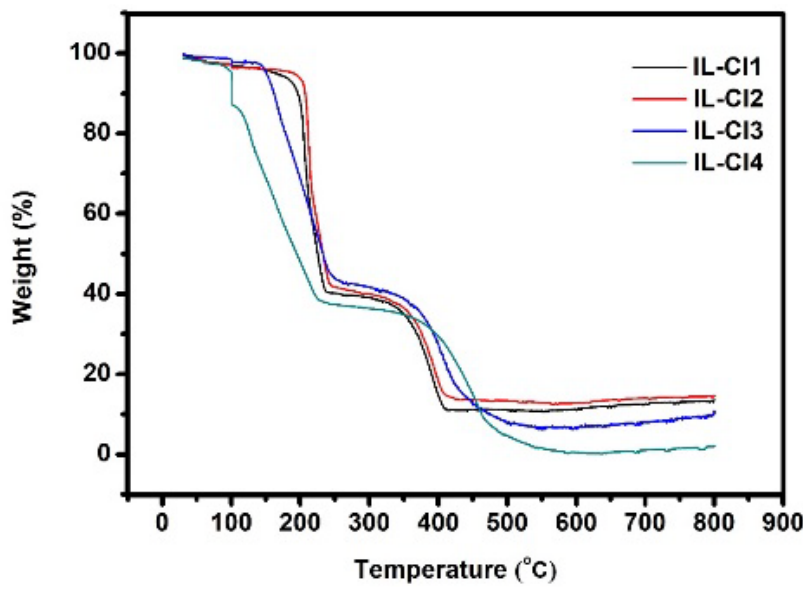
2	IL-Cl2	195	243	410		
3	IL-Cl3	191	250	386		
4	IL-Cl4	216	267	391		
5	IL-Br1	100	276	599	$[CuBr_2^-]$	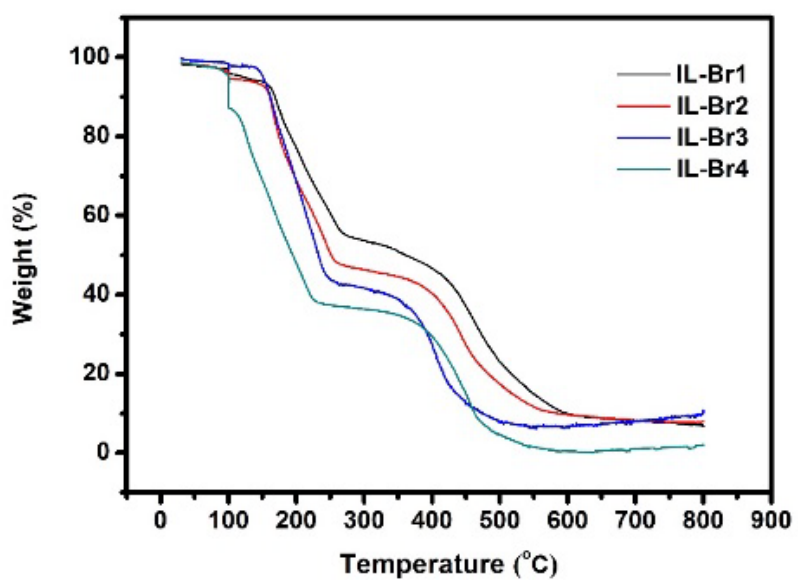
6	IL-Br2	100	270	595		
7	IL-Br3	145	257	467		
8	IL-Br4	100	229	465		
9	IL-I1	100	277	597	$[CuI_2^-]$	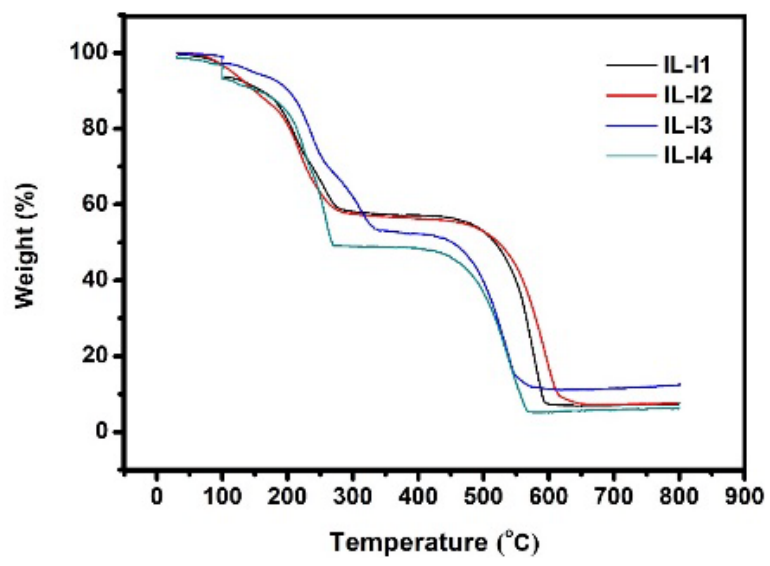
10	IL-I2	-	273	618		
11	IL-I3	100	332	574		
12	IL-I4	99	270	569		

T_d denotes the decomposition temperature. $T_{d,\ 5\%}$, $T_{d,60\%}$, and $T_{d,\ 90\%}$ represent the temperatures at which 5%, 60%, and 90% weight loss occurred, respectively. All measurements were conducted at a heating rate of 5 °C/min.

The thermal stability of the synthesized ionic liquids, evaluated via TGA (Table S2), follows the order: $[CuCl_2^-]$ > $[CuBr_2^-]$ > $[CuI_2^-]$. This trend highlights the significant influence of the halide ligand, where the higher polarizability and metal-halide bond strength of the iodide-based anion enhance thermal robustness. Furthermore, the cationic substituent plays a critical role: while hydrogen (–H) and methyl (–CH_3) groups impart higher stability to $[CuCl_2^-]$ and $[CuBr_2^-]$, the methyl-substituted cation exhibits the most outstanding robustness with the $[CuI_2^-]$ anion. This variation

suggests a synergistic effect between the copper-halide anion and the cationic substituent, likely optimizing the electronic environment to maximize the thermal decomposition temperature.

Reference

1. Solomons, T. W. G.; Fryhle, C. B.; Snyder, S. A. *Organic Chemistry*; Wiley, 2016.

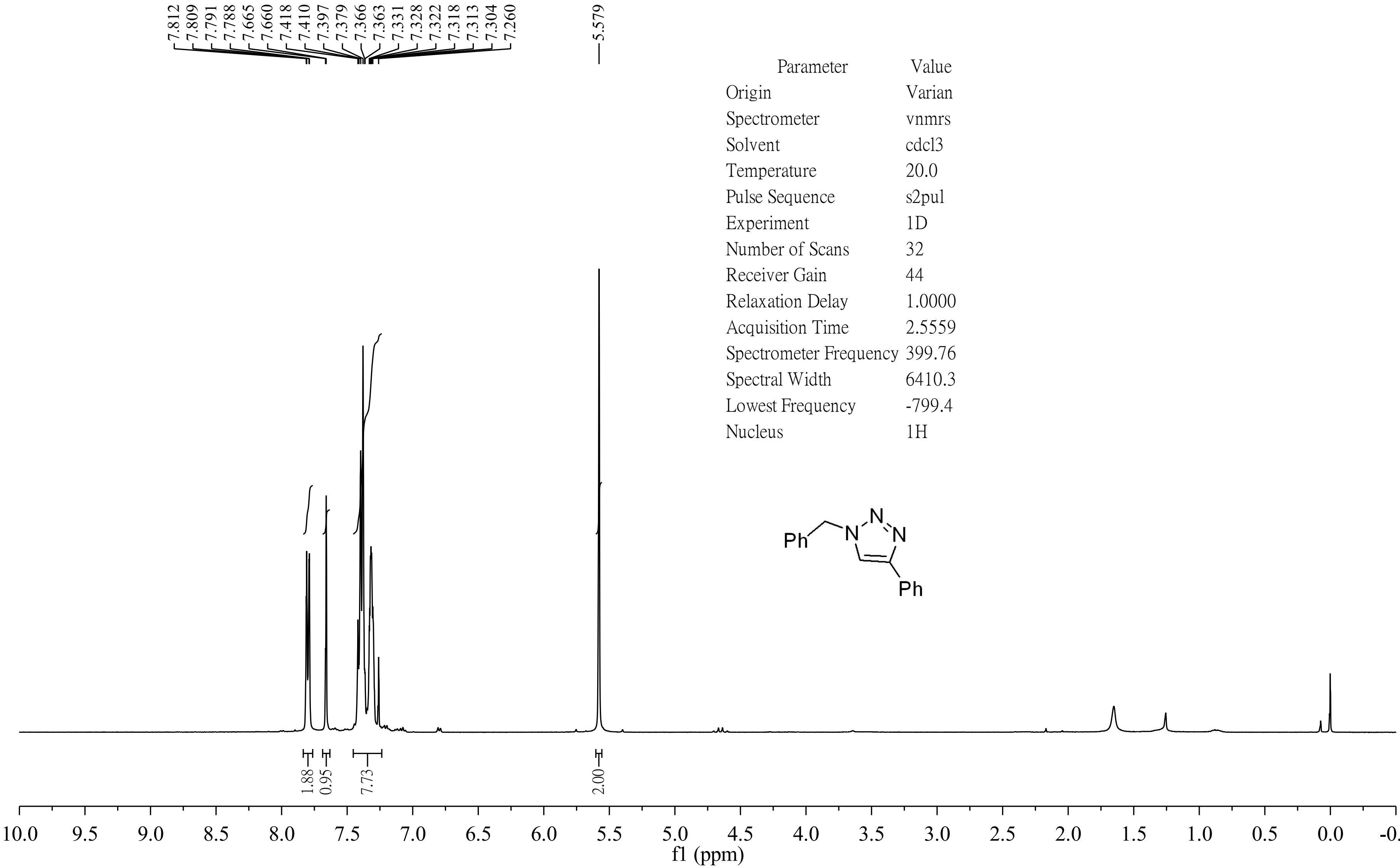

Parameter	Value
Origin	Varian
Spectrometer	vnmrs
Solvent	cdcl3
Temperature	20.0
Pulse Sequence	s2pul
Experiment	1D
Number of Scans	32
Receiver Gain	44
Relaxation Delay	1.0000
Acquisition Time	2.5559
Spectrometer Frequency	399.76
Spectral Width	6410.3
Lowest Frequency	-799.4
Nucleus	1H

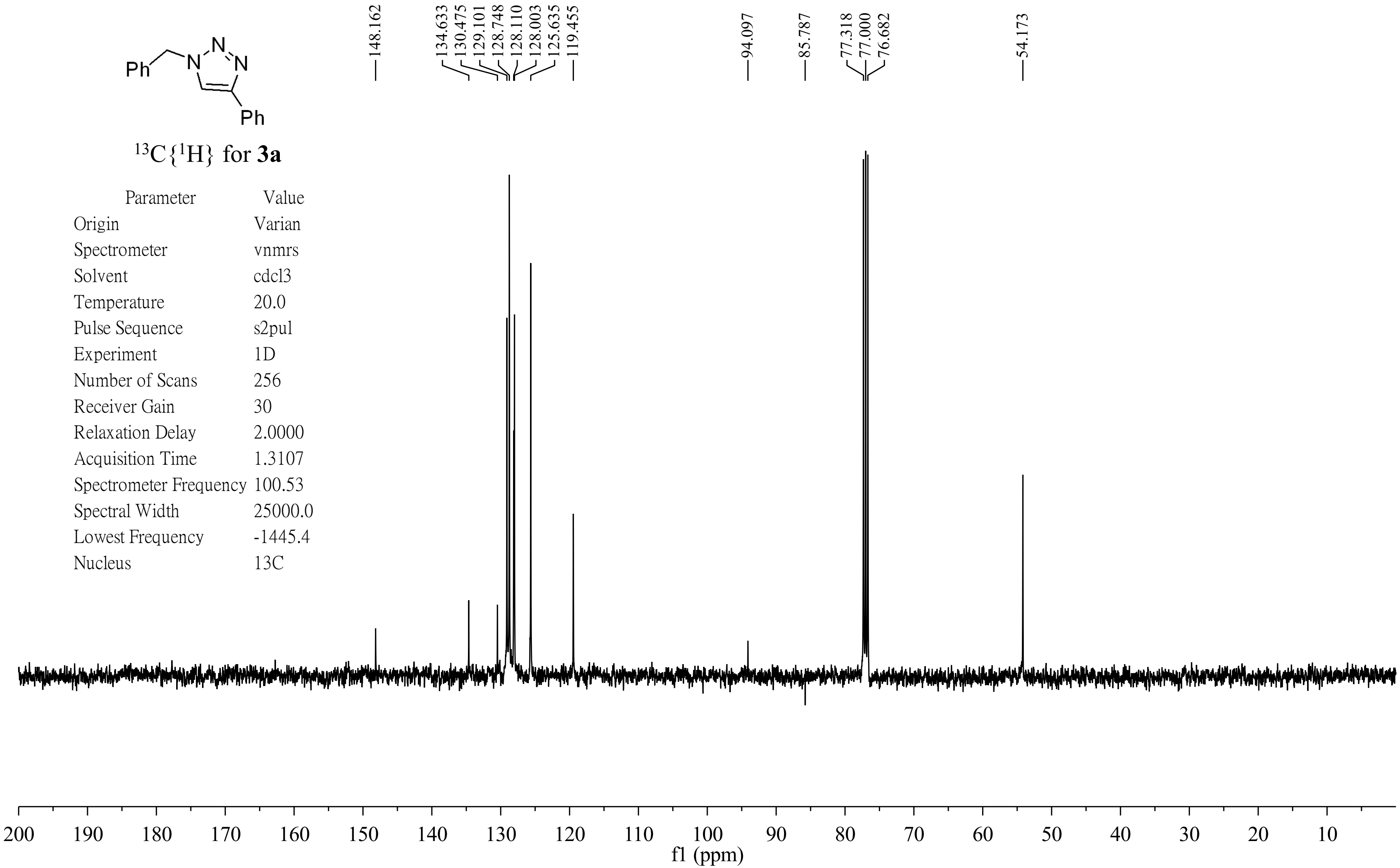
Ph
N
N
N
Ph
13C{1H} for 3a
148.162
134.633
130.475
129.101
128.748
128.110
128.003
125.635
119.455
94.097
85.787
77.318
77.000
76.682
54.173
Parameter Value
Origin Varian
Spectrometer vnmrs
Solvent cdcl3
Temperature 20.0
Pulse Sequence s2pul
Experiment 1D
Number of Scans 256
Receiver Gain 30
Relaxation Delay 2.0000
Acquisition Time 1.3107
Spectrometer Frequency 100.53
Spectral Width 25000.0
Lowest Frequency -1445.4
Nucleus 13C
200 190 180 170 160 150 140 130 120 110 100 90 80 70 60 50 40 30 20 10
f1 (ppm)

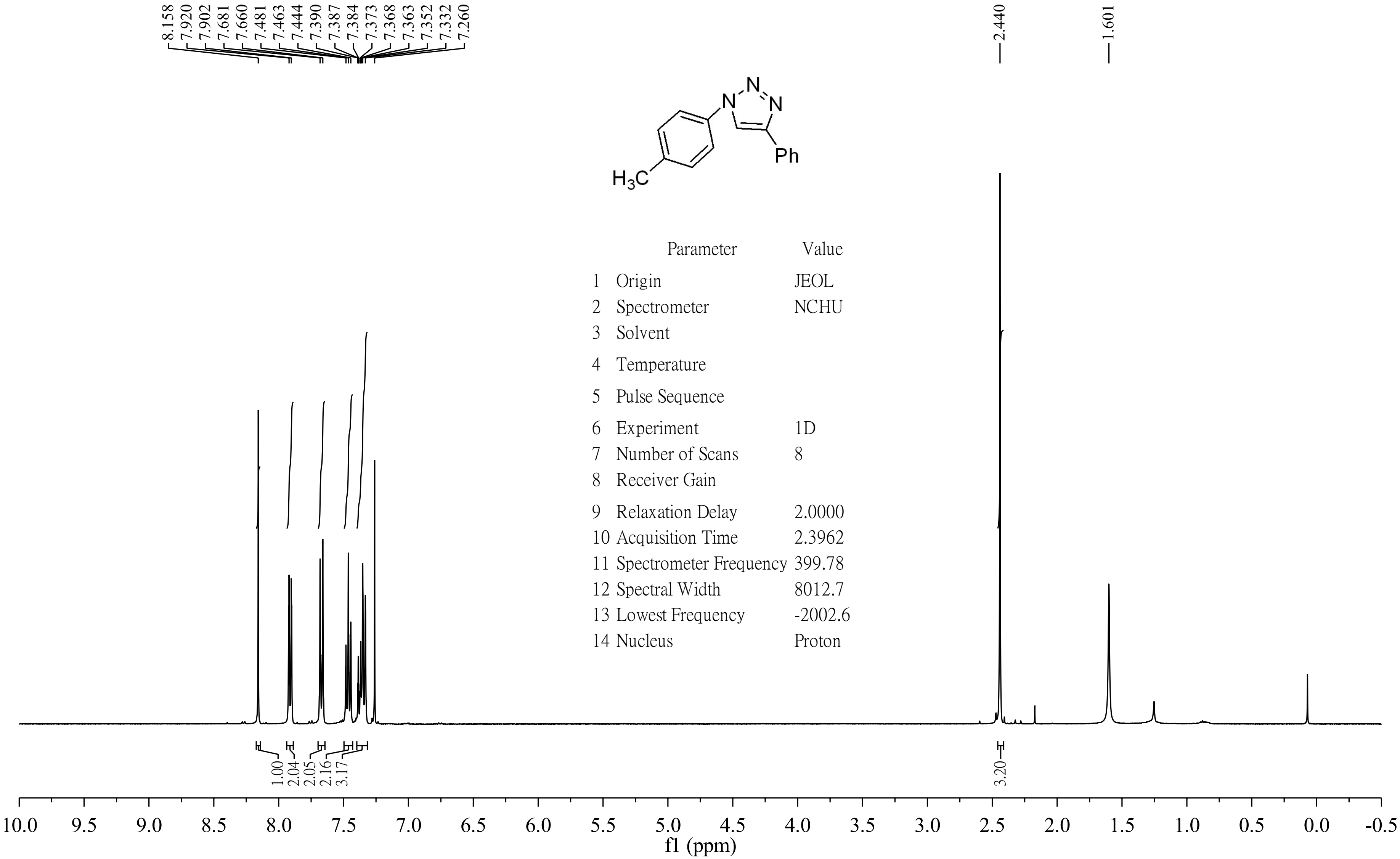
8.158
7.920
7.902
7.681
7.660
7.481
7.463
7.444
7.390
7.387
7.384
7.373
7.368
7.363
7.352
7.332
7.260
2.440
1.601
N
N
N
Ph
H3C
Parameter Value
1 Origin JEOL
2 Spectrometer NCHU
3 Solvent
4 Temperature
5 Pulse Sequence
6 Experiment 1D
7 Number of Scans 8
8 Receiver Gain
9 Relaxation Delay 2.0000
10 Acquisition Time 2.3962
11 Spectrometer Frequency 399.78
12 Spectral Width 8012.7
13 Lowest Frequency -2002.6
14 Nucleus Proton
1.00
2.04
2.05
2.16
3.17
3.20
10.0 9.5 9.0 8.5 8.0 7.5 7.0 6.5 6.0 5.5 5.0 4.5 4.0 3.5 3.0 2.5 2.0 1.5 1.0 0.5 0.0 -0.5
f1 (ppm)

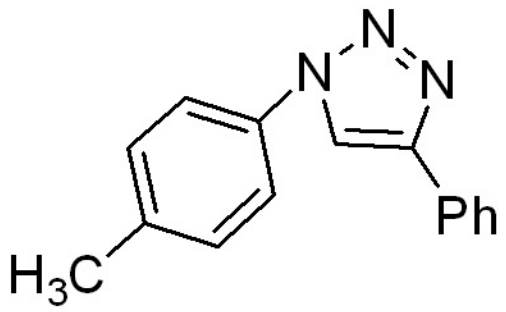

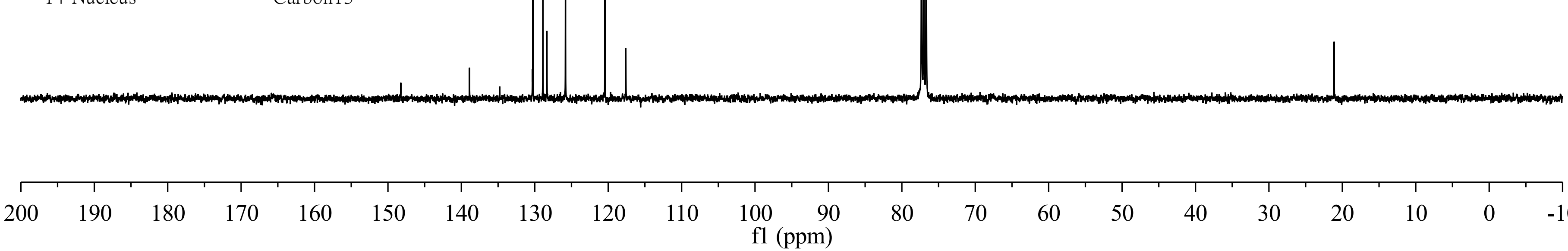

$^{13}C\{^{1}H\}$ for **3b**

	Parameter	Value
1	Origin	JEOL
2	Spectrometer	NCHU
3	Solvent	
4	Temperature	
5	Pulse Sequence	
6	Experiment	1D
7	Number of Scans	1130
8	Receiver Gain	
9	Relaxation Delay	2.0000
10	Acquisition Time	0.7920
11	Spectrometer Frequency	100.53
12	Spectral Width	25252.1
13	Lowest Frequency	-2584.7
14	Nucleus	Carbon13

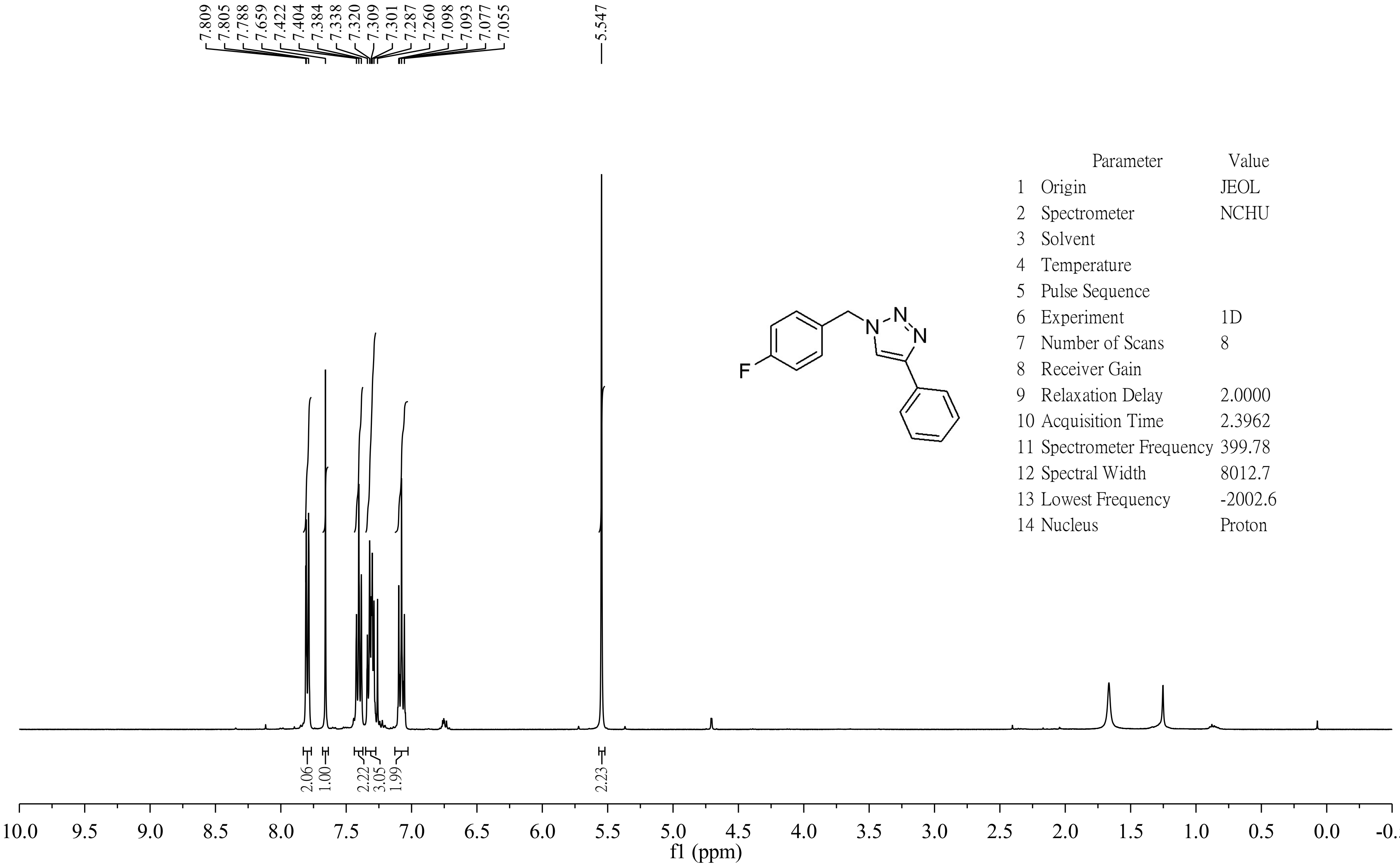
7.809
7.805
7.788
7.659
7.422
7.404
7.384
7.338
7.320
7.309
7.301
7.287
7.260
7.098
7.093
7.077
7.055
5.547
Parameter Value
1 Origin JEOL
2 Spectrometer NCHU
3 Solvent
4 Temperature
5 Pulse Sequence
6 Experiment 1D
7 Number of Scans 8
8 Receiver Gain
9 Relaxation Delay 2.0000
10 Acquisition Time 2.3962
11 Spectrometer Frequency 399.78
12 Spectral Width 8012.7
13 Lowest Frequency -2002.6
14 Nucleus Proton
F
N
N
N
2.06
1.00
2.22
3.05
1.99
2.23
10.0 9.5 9.0 8.5 8.0 7.5 7.0 6.5 6.0 5.5 5.0 4.5 4.0 3.5 3.0 2.5 2.0 1.5 1.0 0.5 0.0 -0.5
f1 (ppm)

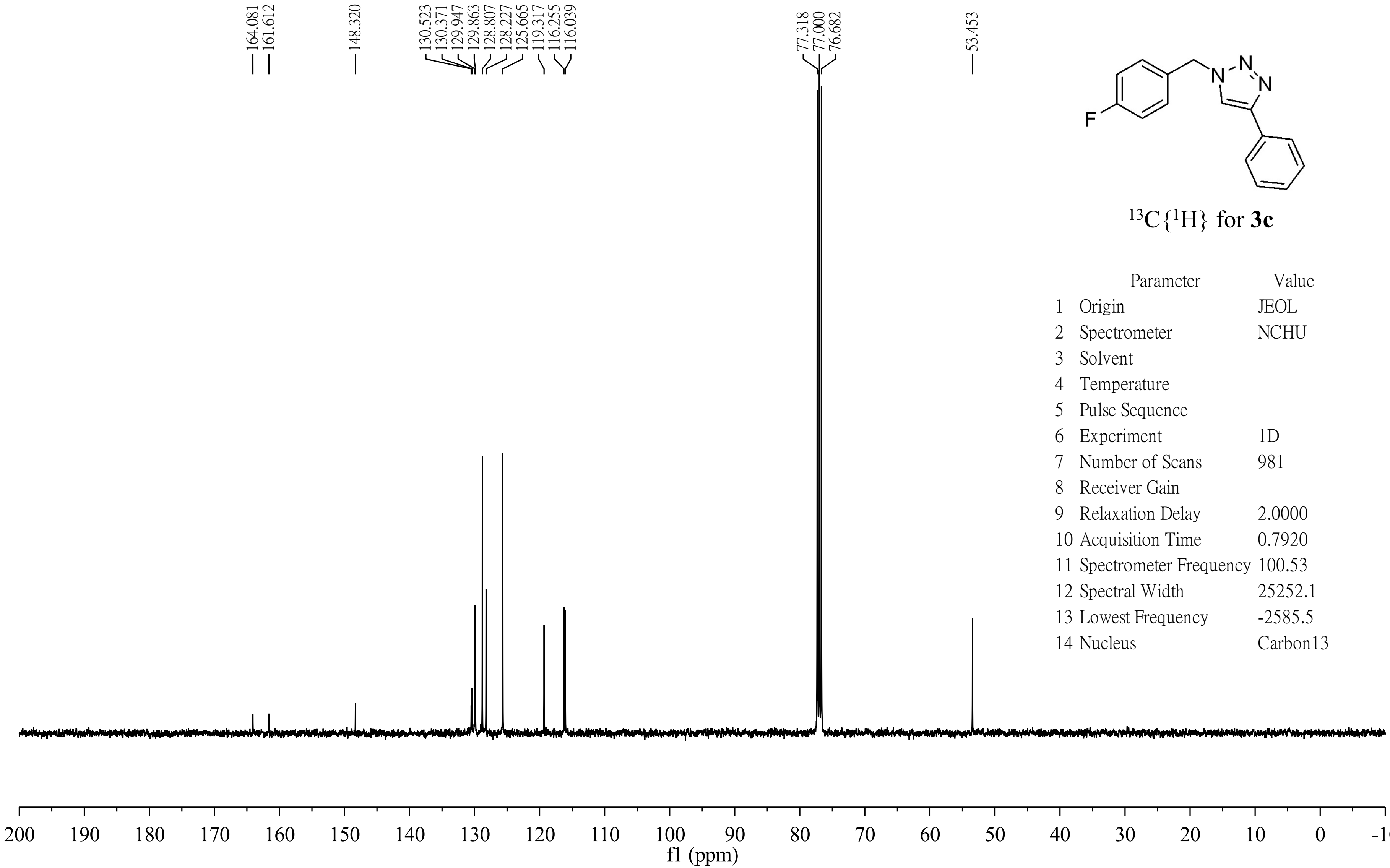

$^{13}C\{^{1}H\}$ for **3c**

	Parameter	Value
1	Origin	JEOL
2	Spectrometer	NCHU
3	Solvent	
4	Temperature	
5	Pulse Sequence	
6	Experiment	1D
7	Number of Scans	981
8	Receiver Gain	
9	Relaxation Delay	2.0000
10	Acquisition Time	0.7920
11	Spectrometer Frequency	100.53
12	Spectral Width	25252.1
13	Lowest Frequency	-2585.5
14	Nucleus	Carbon13

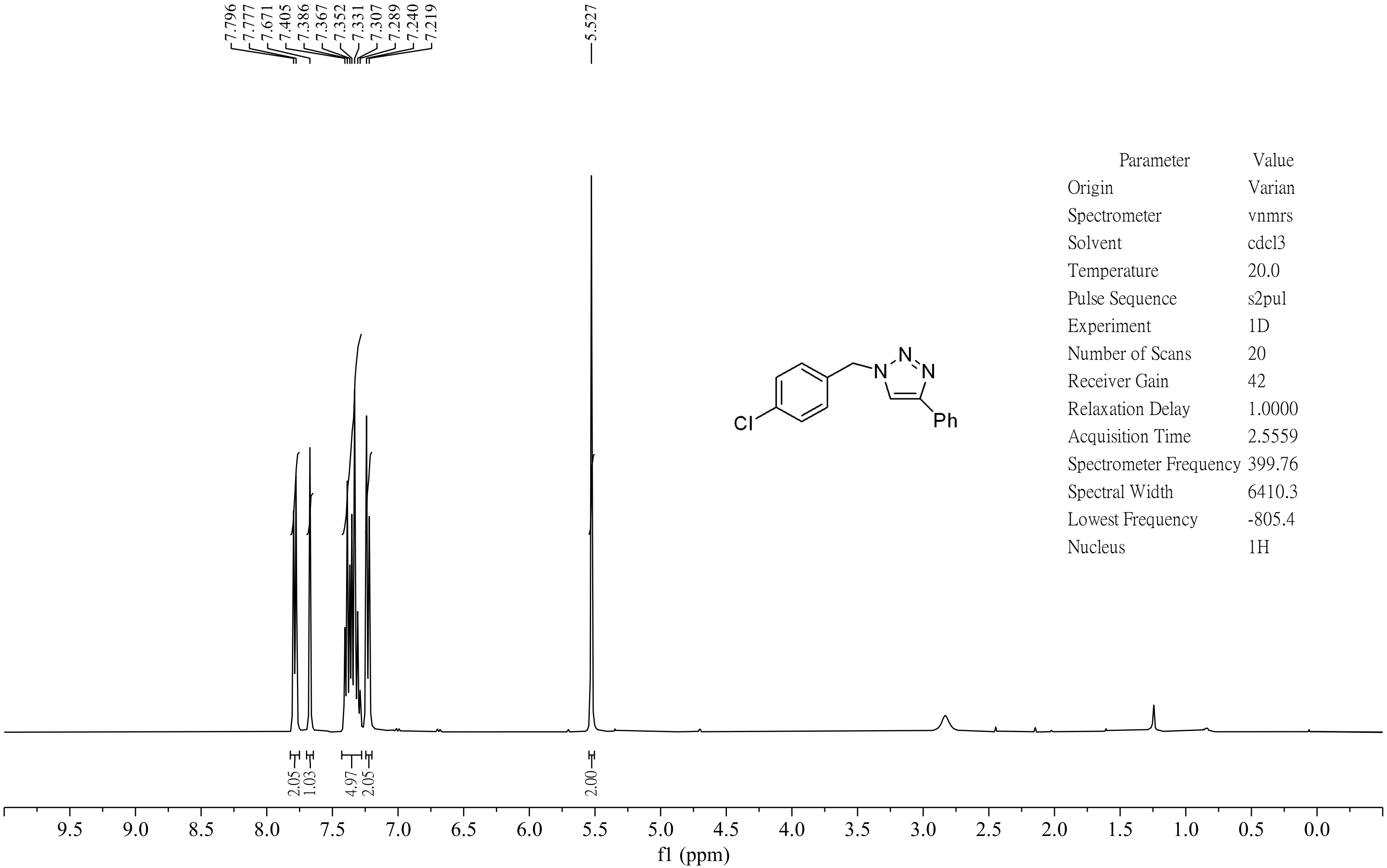

Parameter	Value
Origin	Varian
Spectrometer	vnmrs
Solvent	cdcl3
Temperature	20.0
Pulse Sequence	s2pul
Experiment	1D
Number of Scans	20
Receiver Gain	42
Relaxation Delay	1.0000
Acquisition Time	2.5559
Spectrometer Frequency	399.76
Spectral Width	6410.3
Lowest Frequency	-805.4
Nucleus	1H

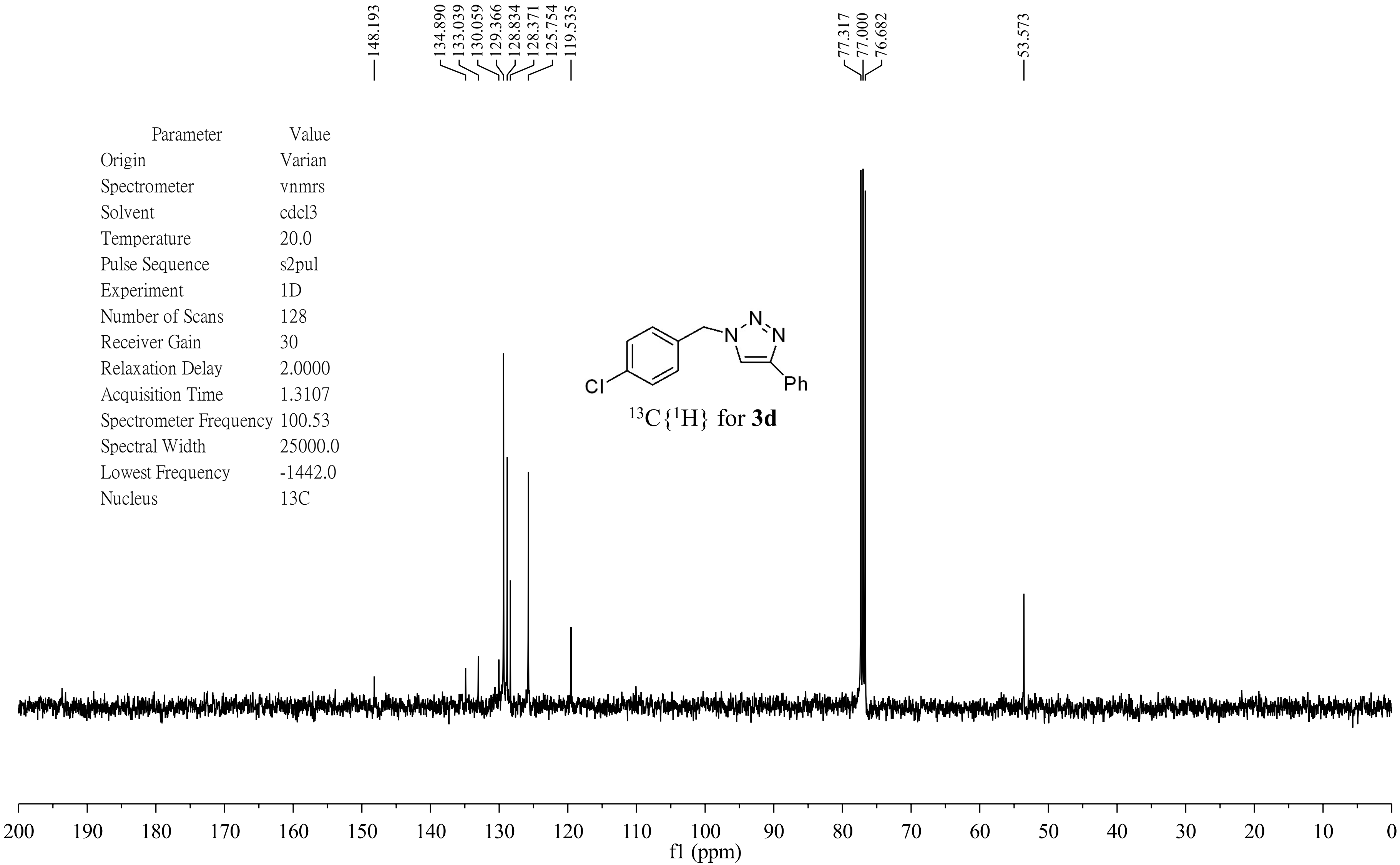

Parameter	Value
Origin	Varian
Spectrometer	vnmrs
Solvent	cdcl3
Temperature	20.0
Pulse Sequence	s2pul
Experiment	1D
Number of Scans	128
Receiver Gain	30
Relaxation Delay	2.0000
Acquisition Time	1.3107
Spectrometer Frequency	100.53
Spectral Width	25000.0
Lowest Frequency	-1442.0
Nucleus	13C

$^{13}C\{^1H\}$ for **3d**

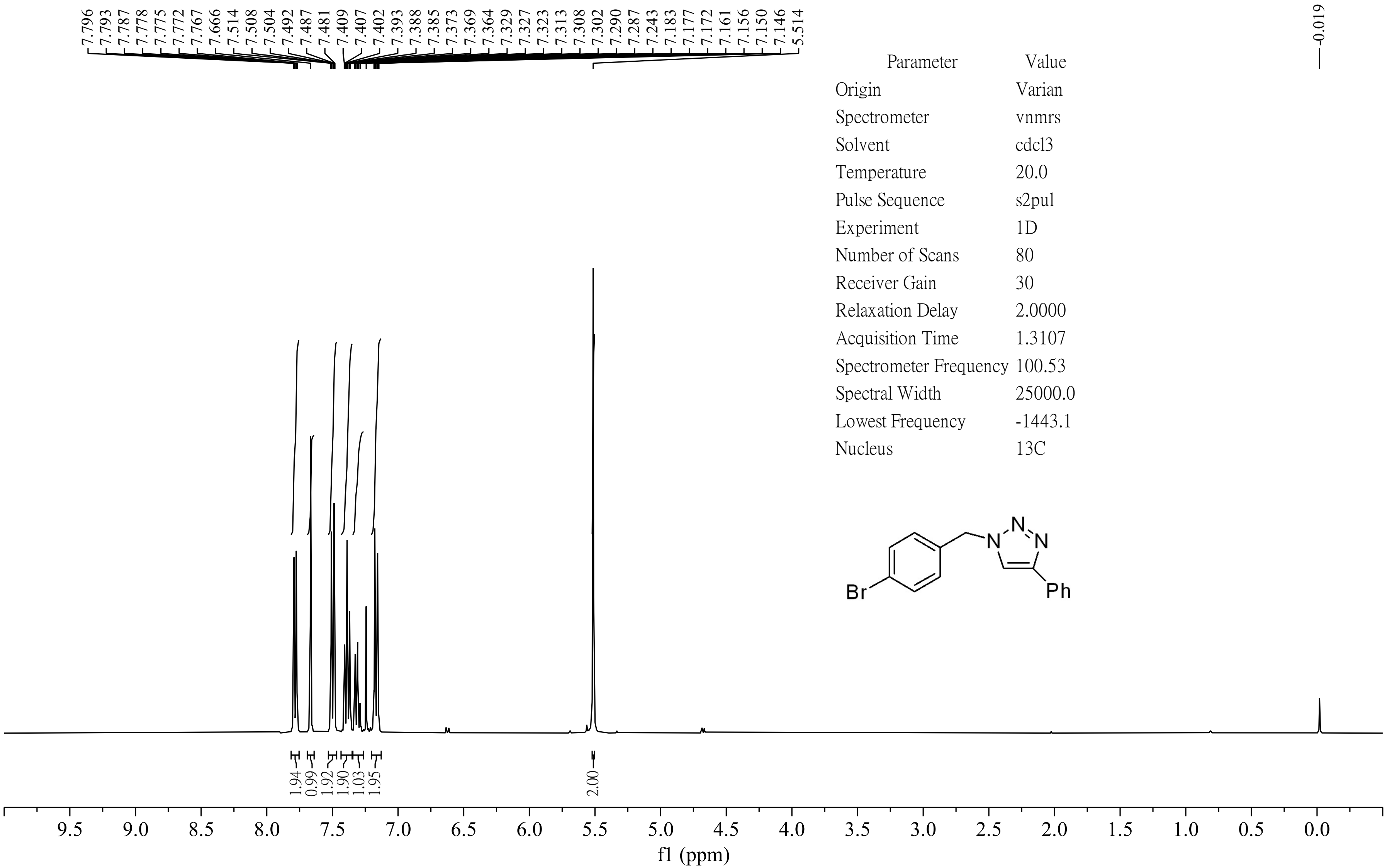
7.796
7.793
7.787
7.778
7.775
7.772
7.767
7.666
7.514
7.508
7.504
7.492
7.487
7.481
7.409
7.407
7.402
7.393
7.388
7.385
7.373
7.369
7.364
7.329
7.327
7.323
7.313
7.308
7.302
7.290
7.287
7.243
7.183
7.177
7.172
7.161
7.156
7.150
7.146
5.514
-0.019
Parameter Value
Origin Varian
Spectrometer vnmrs
Solvent cdcl3
Temperature 20.0
Pulse Sequence s2pul
Experiment 1D
Number of Scans 80
Receiver Gain 30
Relaxation Delay 2.0000
Acquisition Time 1.3107
Spectrometer Frequency 100.53
Spectral Width 25000.0
Lowest Frequency -1443.1
Nucleus 13C
N
N
N
Br
Ph
1.94
0.99
1.92
1.90
1.03
1.95
2.00
9.5 9.0 8.5 8.0 7.5 7.0 6.5 6.0 5.5 5.0 4.5 4.0 3.5 3.0 2.5 2.0 1.5 1.0 0.5 0.0
f1 (ppm)

Parameter	Value
Origin	Varian
Spectrometer	vnmrs
Solvent	cdcl3
Temperature	20.0
Pulse Sequence	s2pul
Experiment	1D
Number of Scans	80
Receiver Gain	30
Relaxation Delay	2.0000
Acquisition Time	1.3107
Spectrometer Frequency	100.53
Spectral Width	25000.0
Lowest Frequency	-1443.1
Nucleus	13C

$^{13}C\{^{1}H\}$ for **3e**

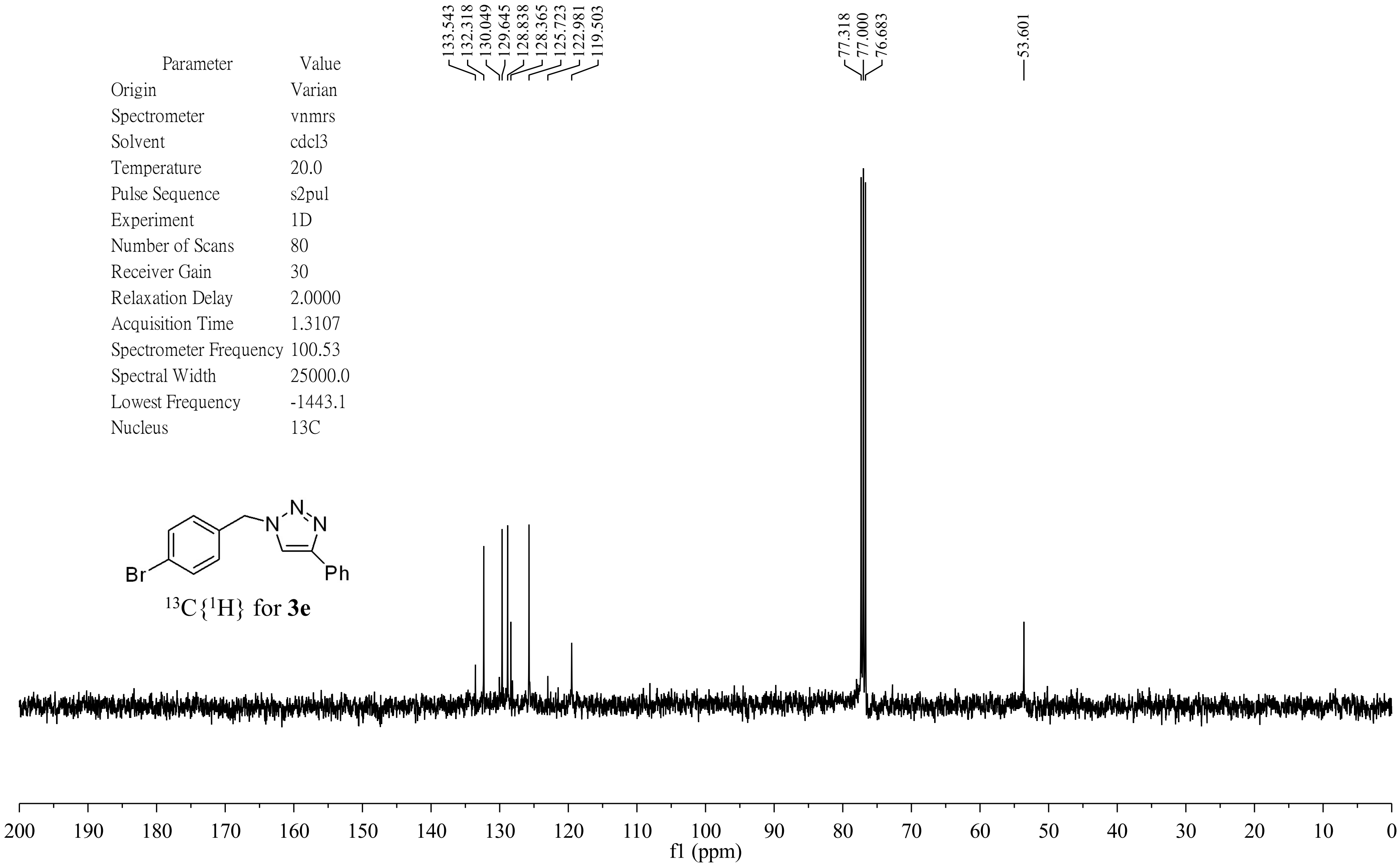

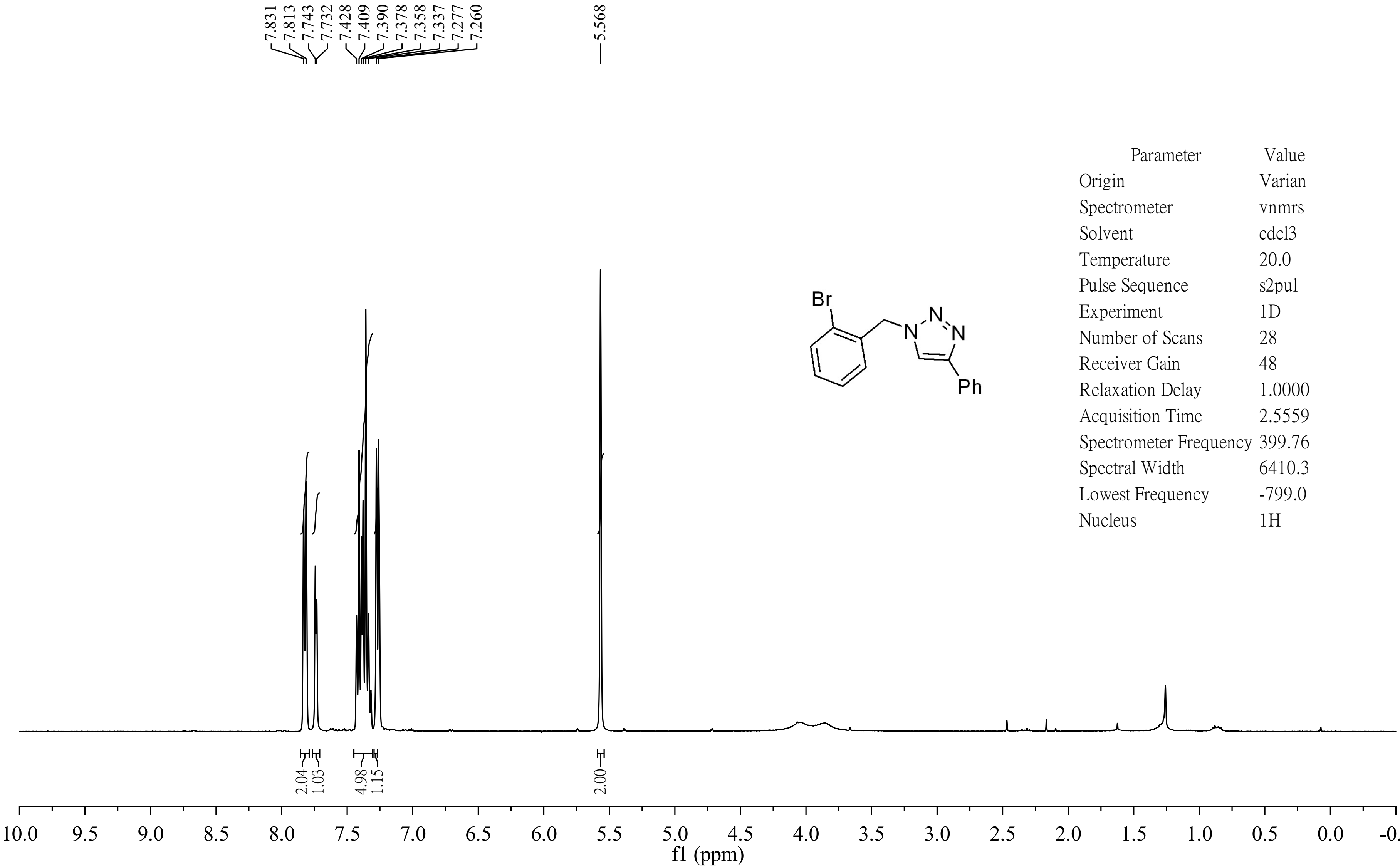
7.831
7.813
7.743
7.732
7.428
7.409
7.390
7.378
7.358
7.337
7.277
7.260
5.568
Br
N
N
N
Ph
Parameter Value
Origin Varian
Spectrometer vnmrs
Solvent cdcl3
Temperature 20.0
Pulse Sequence s2pul
Experiment 1D
Number of Scans 28
Receiver Gain 48
Relaxation Delay 1.0000
Acquisition Time 2.5559
Spectrometer Frequency 399.76
Spectral Width 6410.3
Lowest Frequency -799.0
Nucleus 1H
2.04
1.03
4.98
1.15
2.00
10.0 9.5 9.0 8.5 8.0 7.5 7.0 6.5 6.0 5.5 5.0 4.5 4.0 3.5 3.0 2.5 2.0 1.5 1.0 0.5 0.0 -0.5
f1 (ppm)

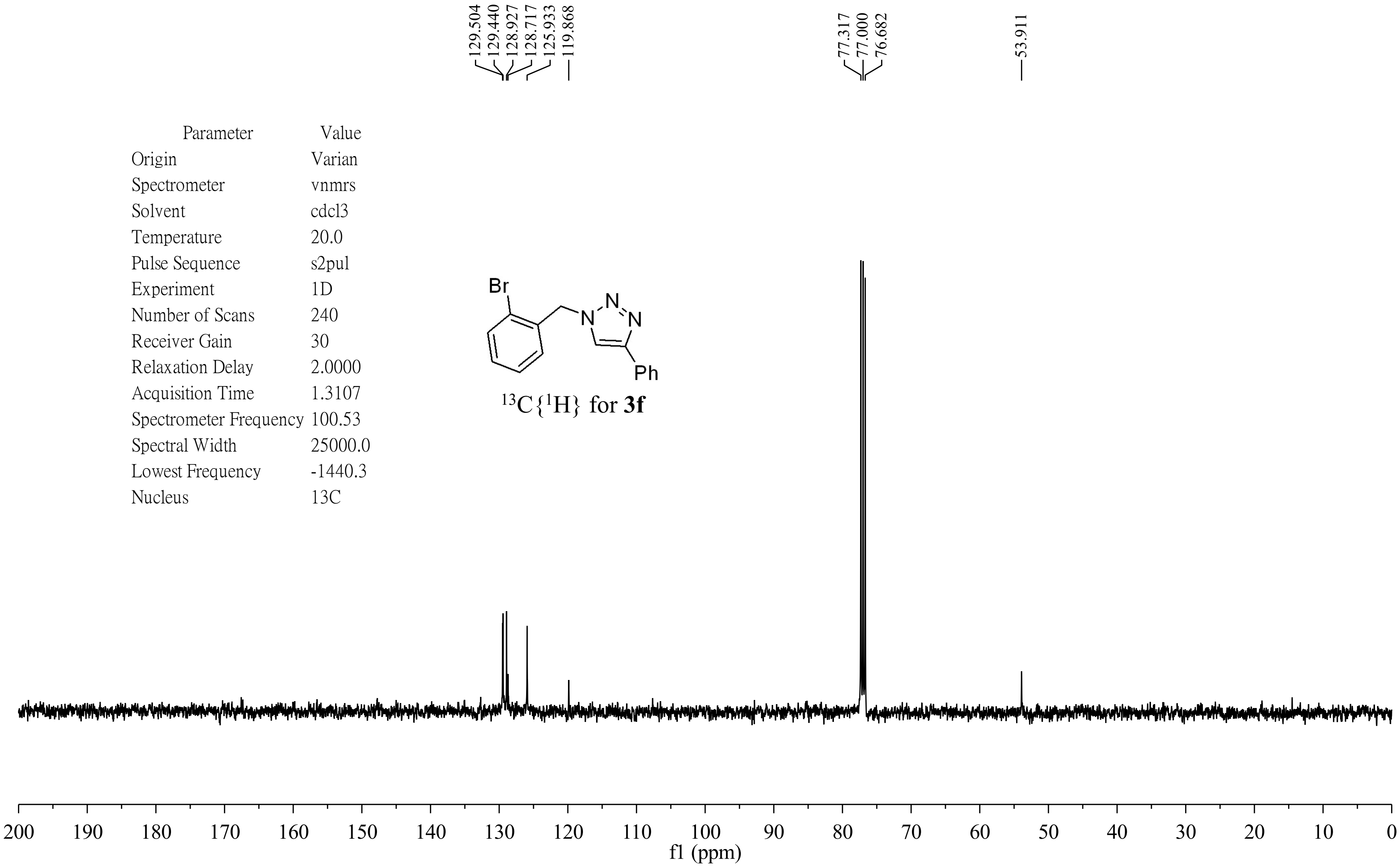

Parameter	Value
Origin	Varian
Spectrometer	vnmrs
Solvent	cdcl3
Temperature	20.0
Pulse Sequence	s2pul
Experiment	1D
Number of Scans	240
Receiver Gain	30
Relaxation Delay	2.0000
Acquisition Time	1.3107
Spectrometer Frequency	100.53
Spectral Width	25000.0
Lowest Frequency	-1440.3
Nucleus	13C

$^{13}C\{^{1}H\}$ for **3f**

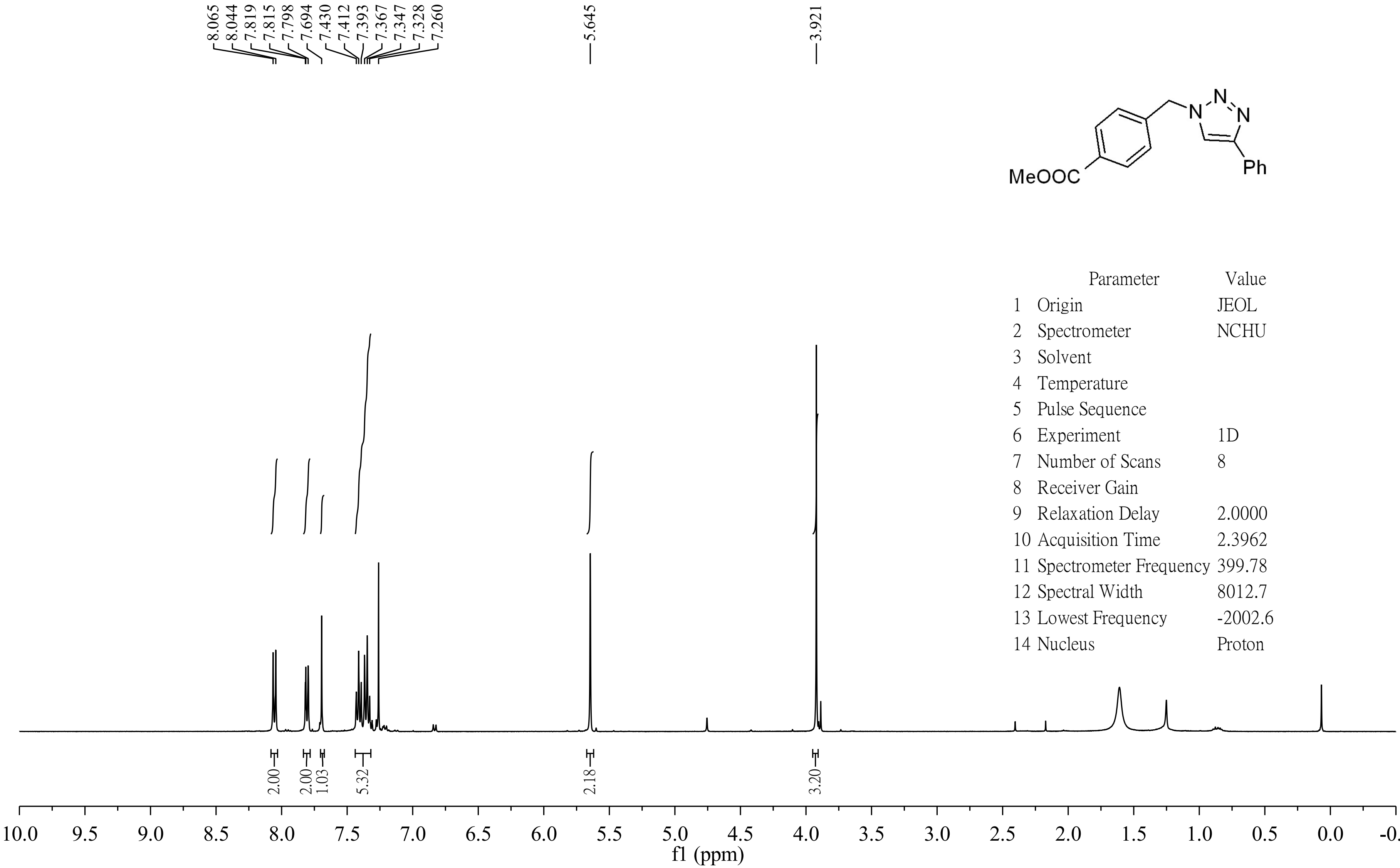

	Parameter	Value
1	Origin	JEOL
2	Spectrometer	NCHU
3	Solvent	
4	Temperature	
5	Pulse Sequence	
6	Experiment	1D
7	Number of Scans	8
8	Receiver Gain	
9	Relaxation Delay	2.0000
10	Acquisition Time	2.3962
11	Spectrometer Frequency	399.78
12	Spectral Width	8012.7
13	Lowest Frequency	-2002.6
14	Nucleus	Proton

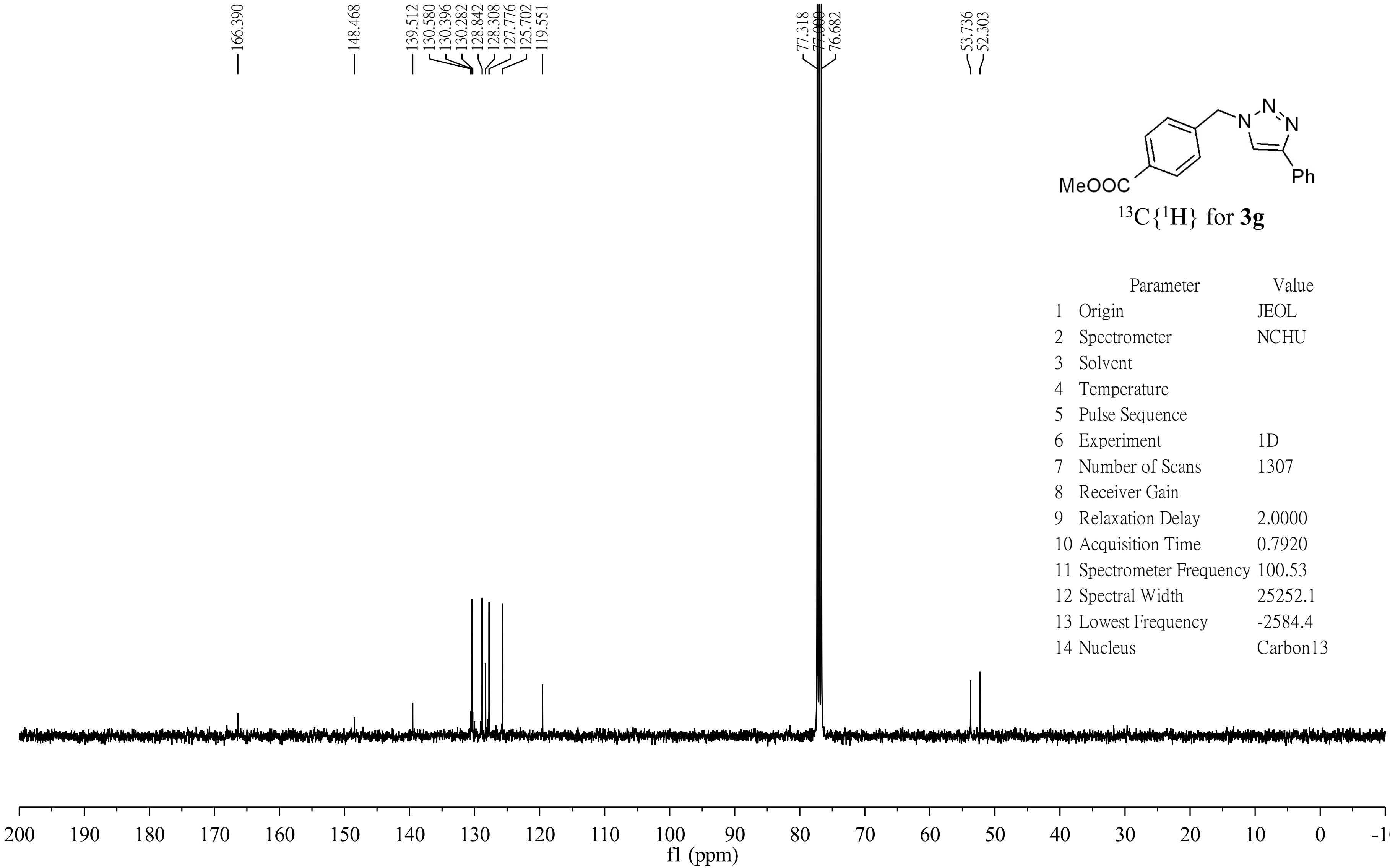

$^{13}C\{^{1}H\}$ for **3g**

	Parameter	Value
1	Origin	JEOL
2	Spectrometer	NCHU
3	Solvent	
4	Temperature	
5	Pulse Sequence	
6	Experiment	1D
7	Number of Scans	1307
8	Receiver Gain	
9	Relaxation Delay	2.0000
10	Acquisition Time	0.7920
11	Spectrometer Frequency	100.53
12	Spectral Width	25252.1
13	Lowest Frequency	-2584.4
14	Nucleus	Carbon13

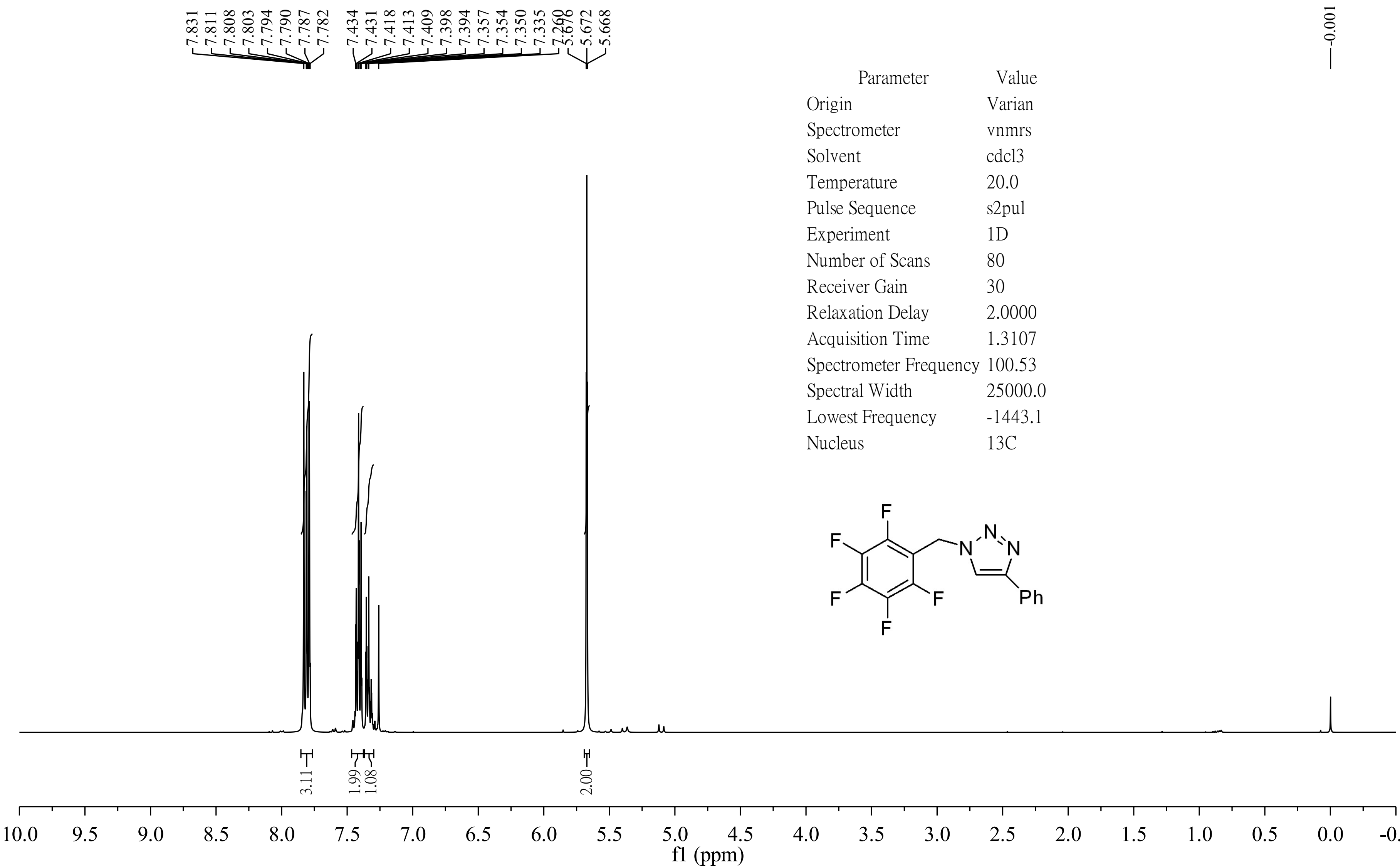

Parameter	Value
Origin	Varian
Spectrometer	vnmrs
Solvent	cdcl3
Temperature	20.0
Pulse Sequence	s2pul
Experiment	1D
Number of Scans	80
Receiver Gain	30
Relaxation Delay	2.0000
Acquisition Time	1.3107
Spectrometer Frequency	100.53
Spectral Width	25000.0
Lowest Frequency	-1443.1
Nucleus	13C

Parameter	Value
Origin	Varian
Spectrometer	vnmrs
Solvent	cdcl3
Temperature	20.0
Pulse Sequence	s2pul
Experiment	1D
Number of Scans	80
Receiver Gain	30
Relaxation Delay	2.0000
Acquisition Time	1.3107
Spectrometer Frequency	100.53
Spectral Width	25000.0
Lowest Frequency	-1443.1
Nucleus	13C

$^{13}C\{^{1}H\}$ for **3h**

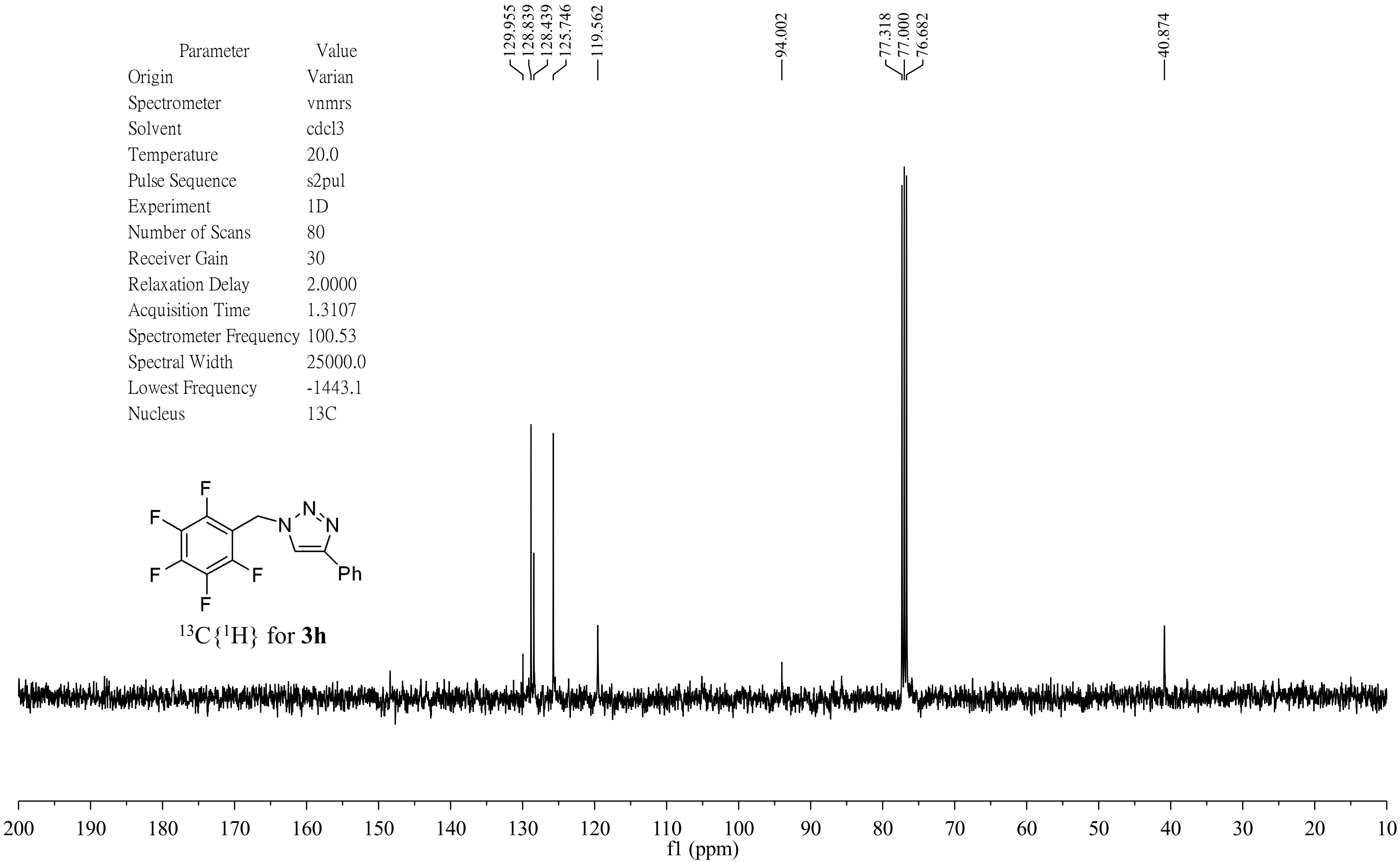

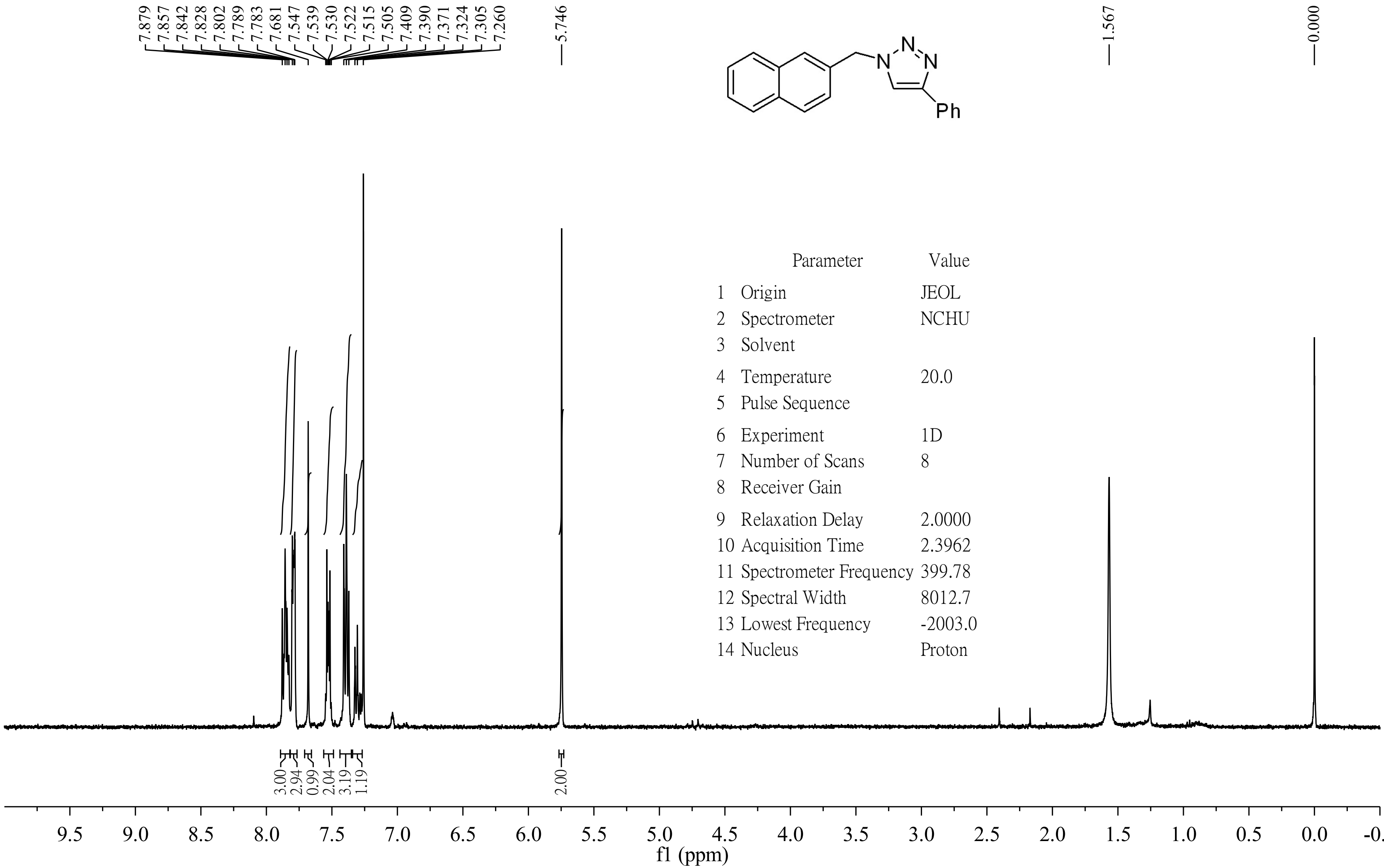
7.879
7.857
7.842
7.828
7.802
7.789
7.783
7.681
7.547
7.539
7.530
7.522
7.515
7.505
7.409
7.390
7.371
7.324
7.305
7.260
5.746
1.567
0.000
N
N
N
Ph
Parameter Value
1 Origin JEOL
2 Spectrometer NCHU
3 Solvent
4 Temperature 20.0
5 Pulse Sequence
6 Experiment 1D
7 Number of Scans 8
8 Receiver Gain
9 Relaxation Delay 2.0000
10 Acquisition Time 2.3962
11 Spectrometer Frequency 399.78
12 Spectral Width 8012.7
13 Lowest Frequency -2003.0
14 Nucleus Proton
3.00
2.94
0.99
2.04
3.19
1.19
2.00
9.5 9.0 8.5 8.0 7.5 7.0 6.5 6.0 5.5 5.0 4.5 4.0 3.5 3.0 2.5 2.0 1.5 1.0 0.5 0.0 -0.5
f1 (ppm)

Parameter	Value
Origin	Varian
Spectrometer	vnmrs
Solvent	cdcl3
Temperature	20.0
Pulse Sequence	s2pul
Experiment	1D
Number of Scans	80
Receiver Gain	30
Relaxation Delay	2.0000
Acquisition Time	1.3107
Spectrometer Frequency	100.53
Spectral Width	25000.0
Lowest Frequency	-1443.1
Nucleus	13C

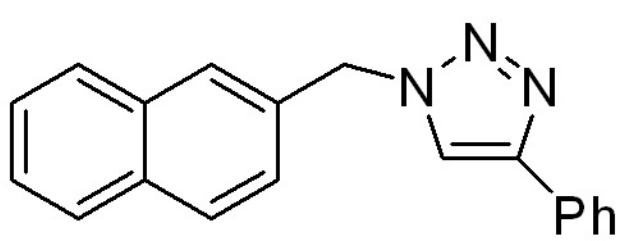

$^{13}C\{^1H\}$ for **3i**

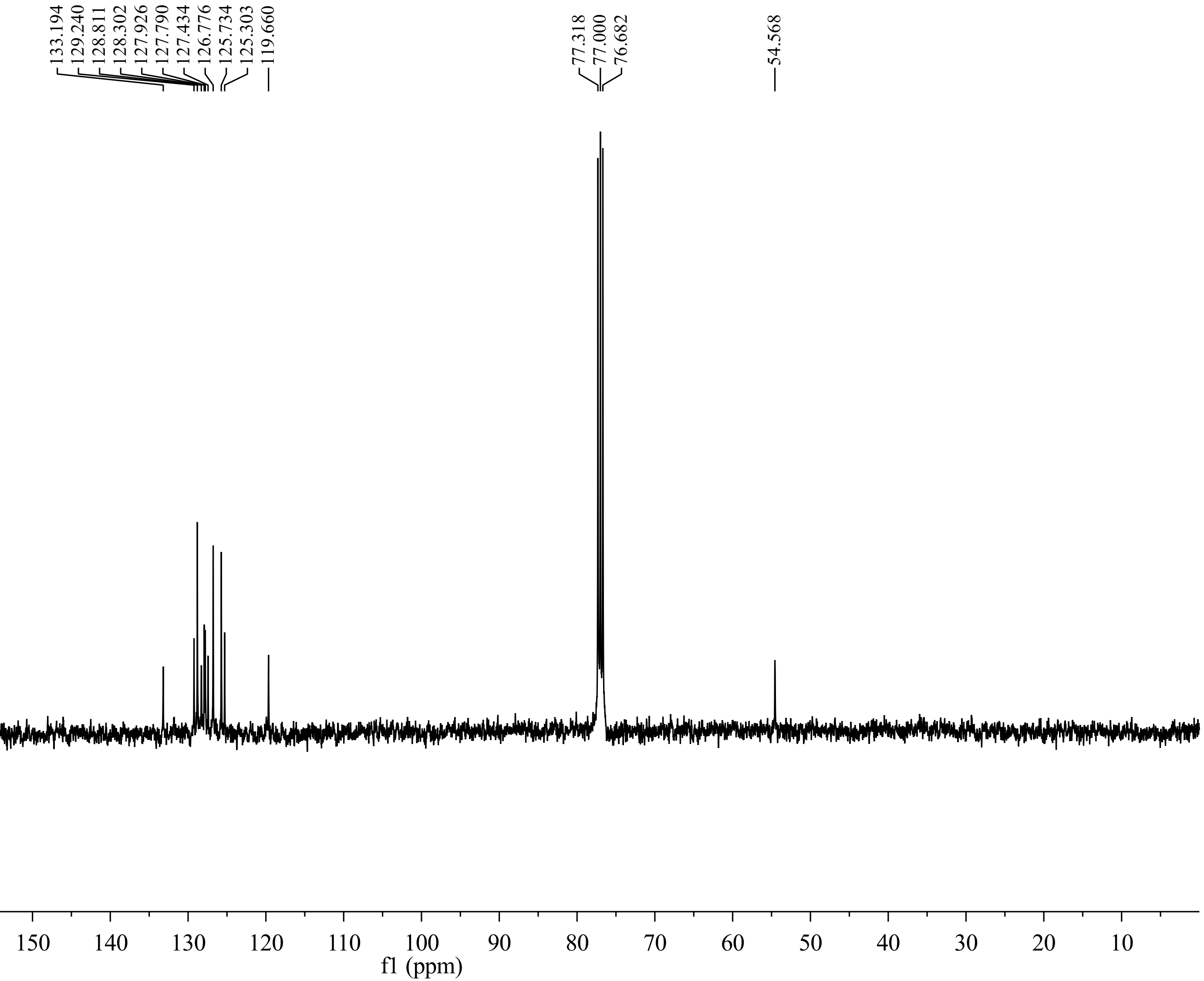

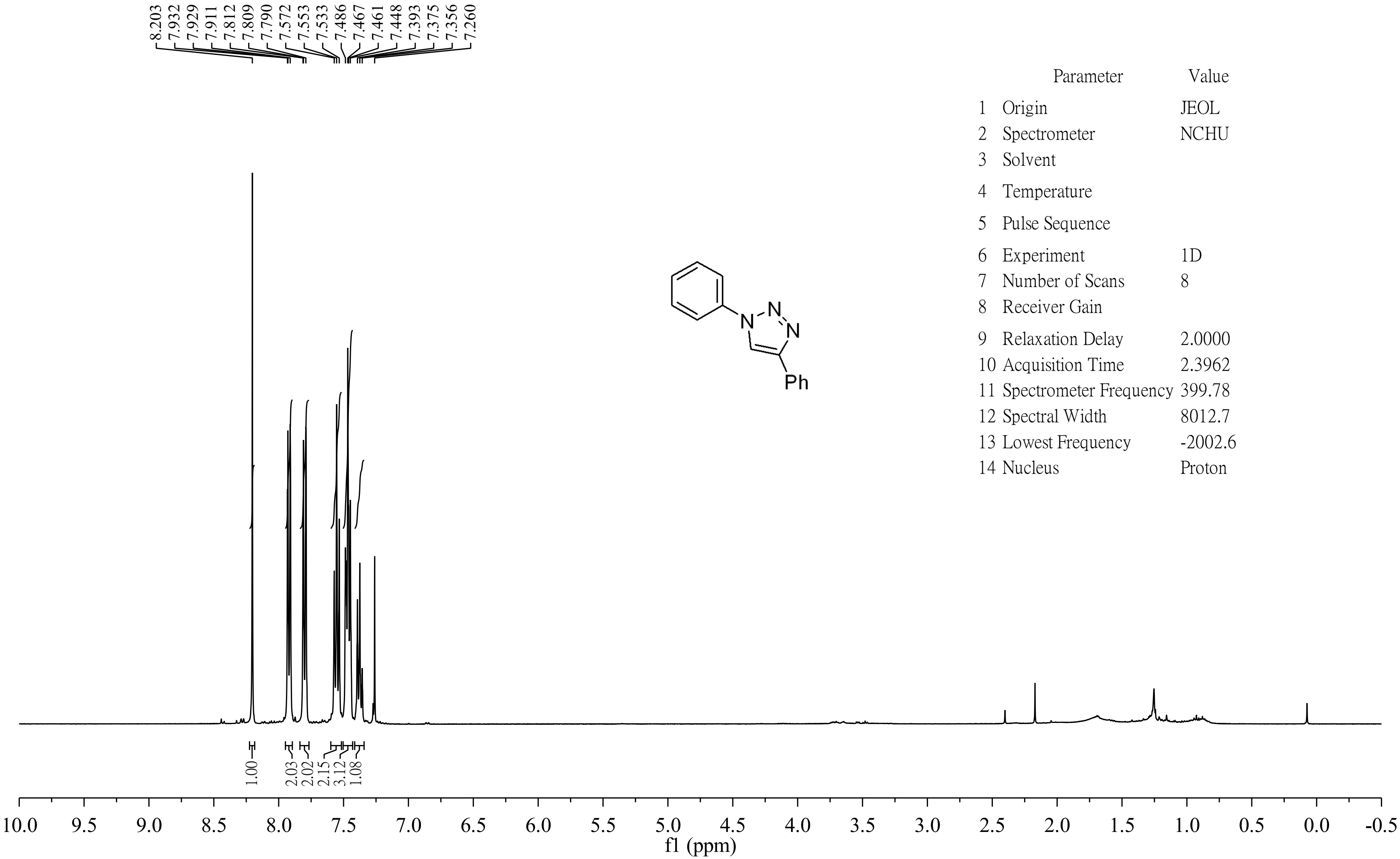

	Parameter	Value
1	Origin	JEOL
2	Spectrometer	NCHU
3	Solvent	
4	Temperature	
5	Pulse Sequence	
6	Experiment	1D
7	Number of Scans	8
8	Receiver Gain	
9	Relaxation Delay	2.0000
10	Acquisition Time	2.3962
11	Spectrometer Frequency	399.78
12	Spectral Width	8012.7
13	Lowest Frequency	-2002.6
14	Nucleus	Proton

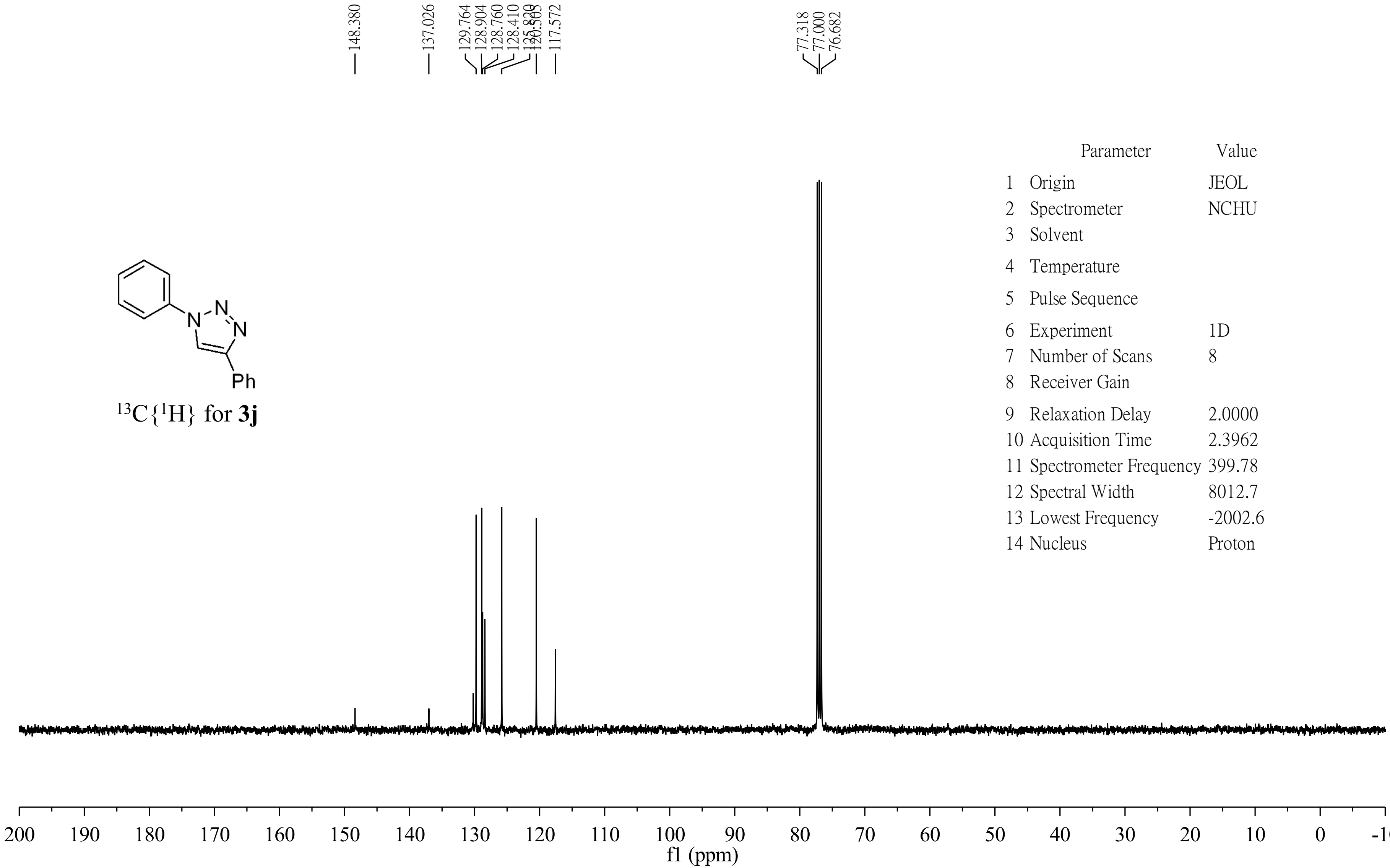

$^{13}C\{^{1}H\}$ for **3j**

	Parameter	Value
1	Origin	JEOL
2	Spectrometer	NCHU
3	Solvent	
4	Temperature	
5	Pulse Sequence	
6	Experiment	1D
7	Number of Scans	8
8	Receiver Gain	
9	Relaxation Delay	2.0000
10	Acquisition Time	2.3962
11	Spectrometer Frequency	399.78
12	Spectral Width	8012.7
13	Lowest Frequency	-2002.6
14	Nucleus	Proton

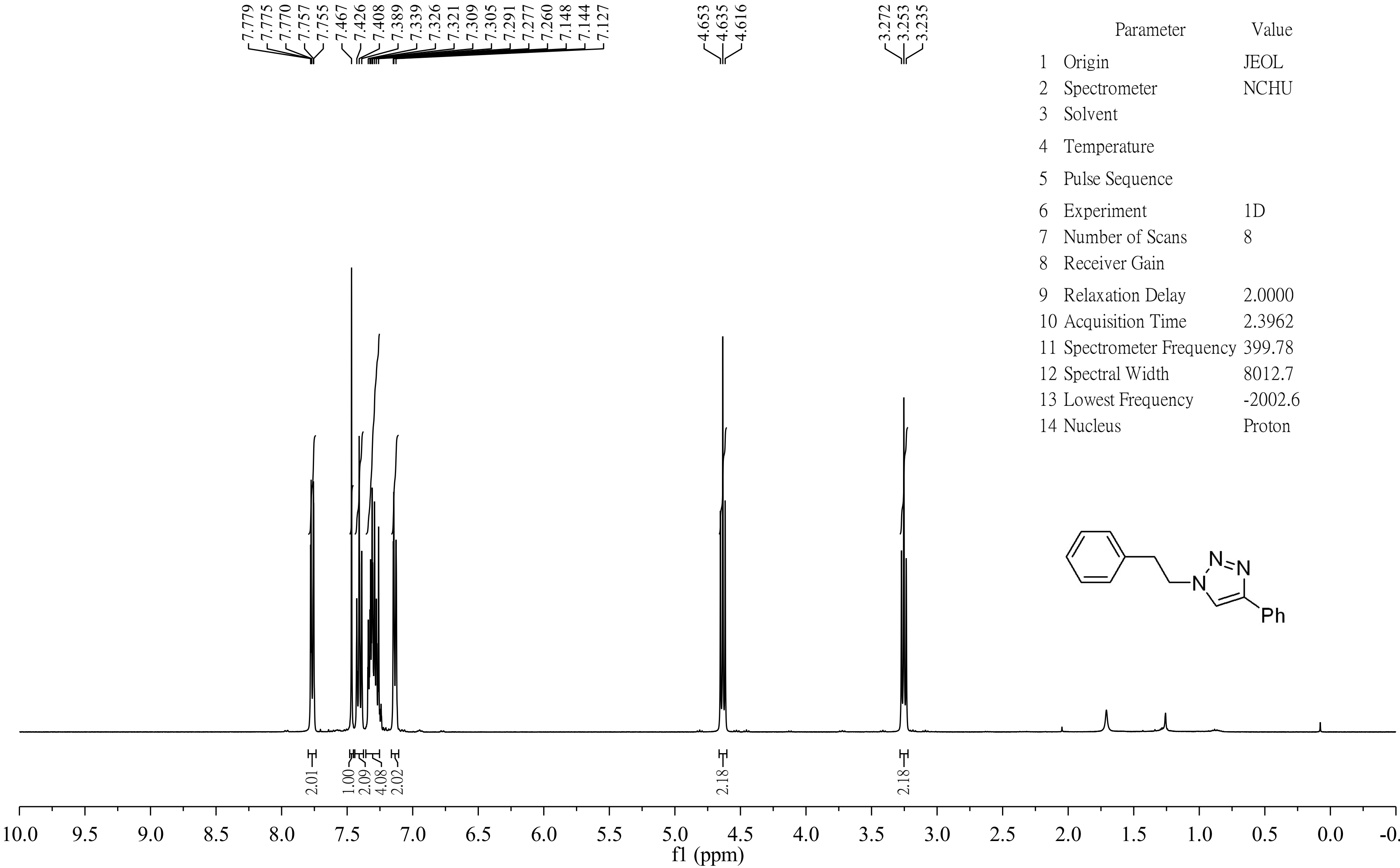

	Parameter	Value
1	Origin	JEOL
2	Spectrometer	NCHU
3	Solvent	
4	Temperature	
5	Pulse Sequence	
6	Experiment	1D
7	Number of Scans	8
8	Receiver Gain	
9	Relaxation Delay	2.0000
10	Acquisition Time	2.3962
11	Spectrometer Frequency	399.78
12	Spectral Width	8012.7
13	Lowest Frequency	-2002.6
14	Nucleus	Proton

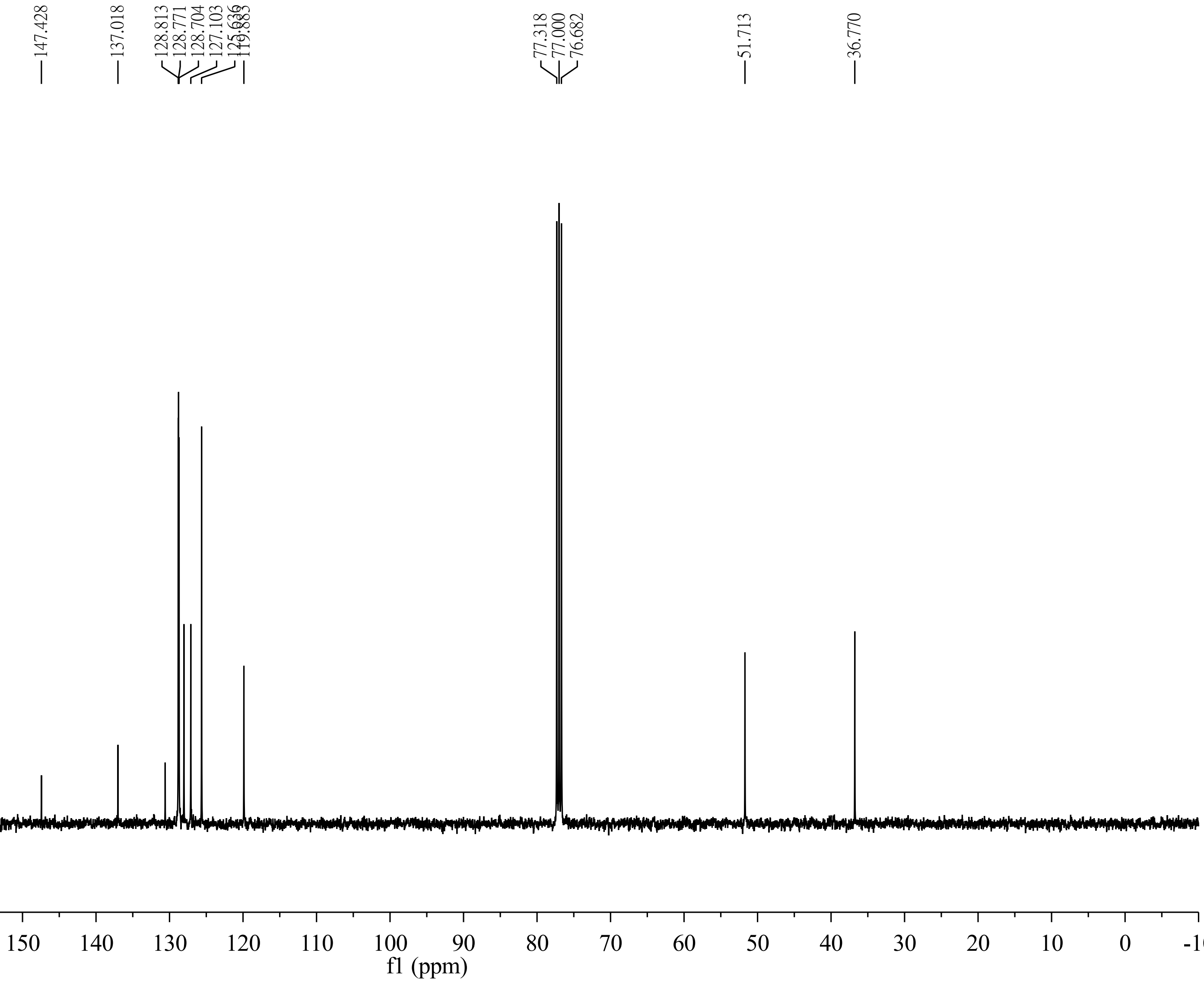

	Parameter	Value
1	Origin	JEOL
2	Spectrometer	NCHU
3	Solvent	
4	Temperature	
5	Pulse Sequence	
6	Experiment	1D
7	Number of Scans	317
8	Receiver Gain	
9	Relaxation Delay	2.0000
10	Acquisition Time	0.7920
11	Spectrometer Frequency	100.53
12	Spectral Width	25252.1
13	Lowest Frequency	-2587.4
14	Nucleus	Carbon13

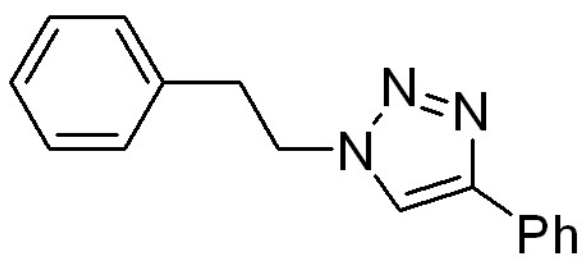

$^{13}C\{^{1}H\}$ for **3k**

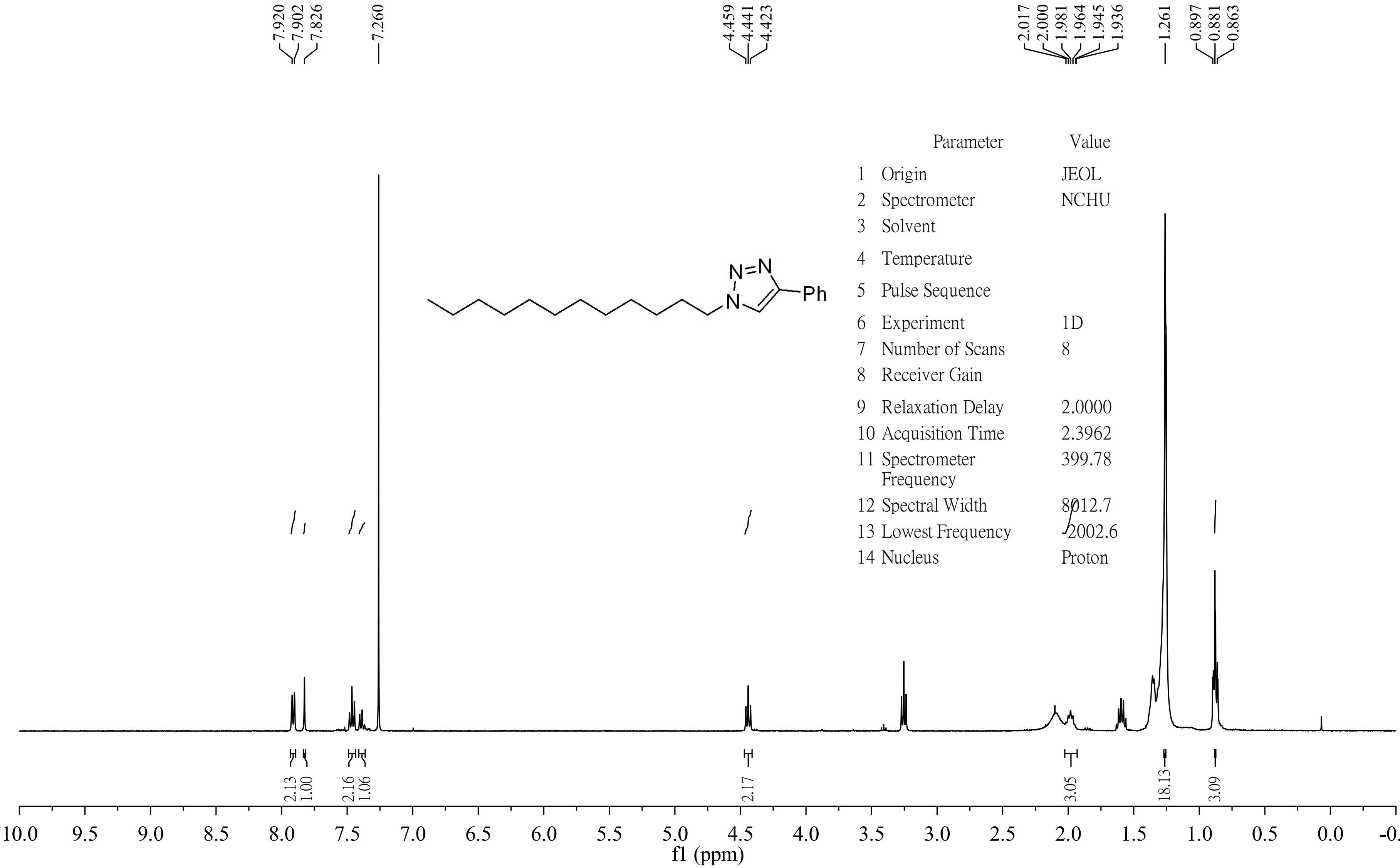
7.920
7.902
7.826
7.260
4.459
4.441
4.423
2.017
2.000
1.981
1.964
1.945
1.936
1.261
0.897
0.881
0.863
N=N
N
Ph
Parameter Value
1 Origin JEOL
2 Spectrometer NCHU
3 Solvent
4 Temperature
5 Pulse Sequence
6 Experiment 1D
7 Number of Scans 8
8 Receiver Gain
9 Relaxation Delay 2.0000
10 Acquisition Time 2.3962
11 Spectrometer Frequency 399.78
12 Spectral Width 8012.7
13 Lowest Frequency -2002.6
14 Nucleus Proton
2.13
1.00
2.16
1.06
2.17
3.05
18.13
3.09
10.0 9.5 9.0 8.5 8.0 7.5 7.0 6.5 6.0 5.5 5.0 4.5 4.0 3.5 3.0 2.5 2.0 1.5 1.0 0.5 0.0 -0.5
f1 (ppm)

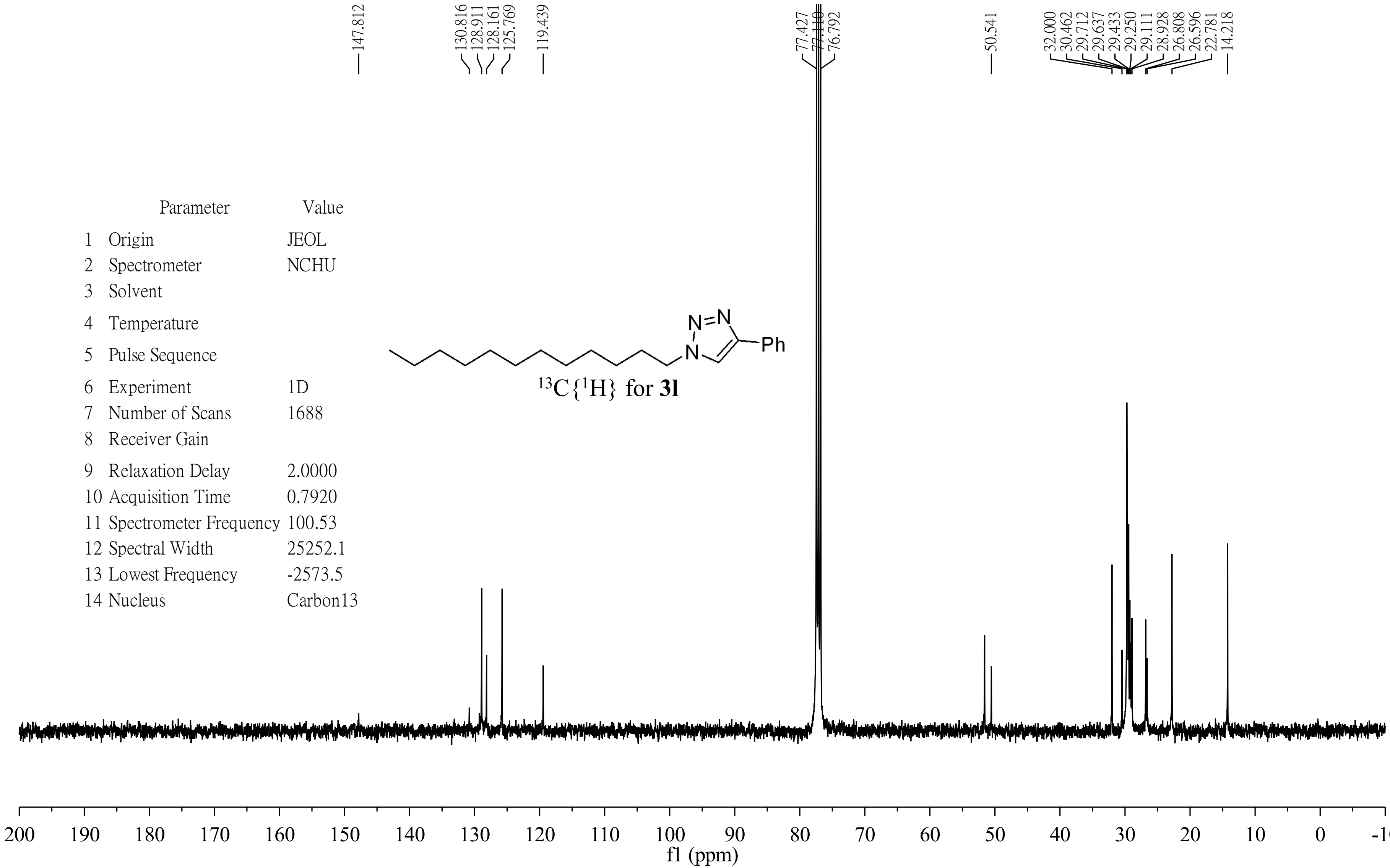

$^{13}C\{^{1}H\}$ for **3l**

	Parameter	Value
1	Origin	JEOL
2	Spectrometer	NCHU
3	Solvent	
4	Temperature	
5	Pulse Sequence	
6	Experiment	1D
7	Number of Scans	1688
8	Receiver Gain	
9	Relaxation Delay	2.0000
10	Acquisition Time	0.7920
11	Spectrometer Frequency	100.53
12	Spectral Width	25252.1
13	Lowest Frequency	-2573.5
14	Nucleus	Carbon13
